# Surface-Active Amidequats with an Alkoxymethyl Substituent: Synthesis, Analysis, and Preliminary Evaluation as Potential Emulsifiers and Substitutes for Conventional Surfactants

**DOI:** 10.1021/acs.langmuir.4c04150

**Published:** 2025-04-16

**Authors:** Anna Syguda, Marta Wojcieszak, Sylwia Zięba, Adam Mizera, Andrzej Łapiński, Jacek Różański, Alicja Putowska, Agnieszka Marcinkowska, Adam Grzywaczyk, Ewa Kaczorek, Katarzyna Materna

**Affiliations:** † Institute of Chemical Technology and Engineering, Faculty of Chemical Technology, Poznan University of Technology, Berdychowo 4, Poznan 60-965, Poland; ‡ Institute of Molecular Physics, Polish Academy of Sciences, M. Smoluchowskiego 17, Poznan 60-179, Poland

## Abstract

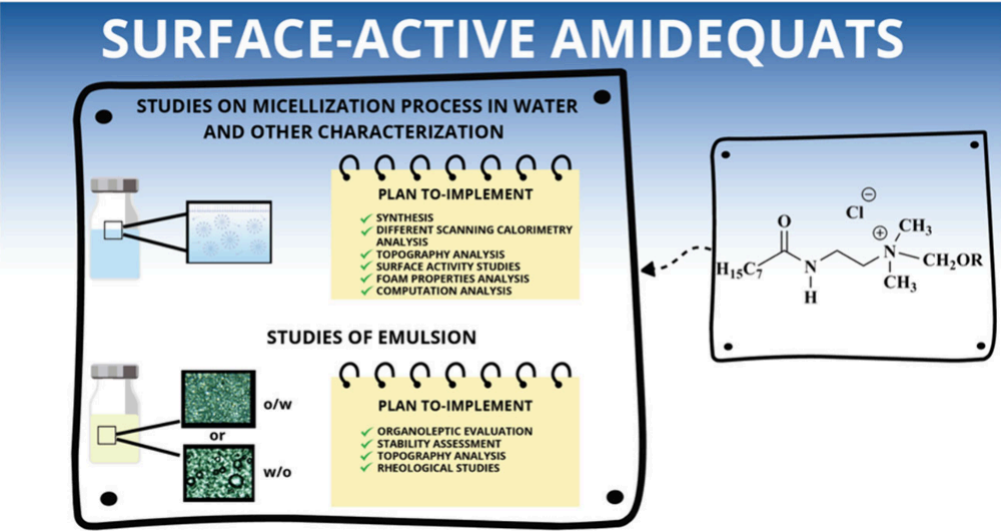

Amidequats have attracted considerable attention in research; however, their surface activity remains largely unknown, and their potential as emulsifiers is still unexplored. In this work, series of novel surface-active amidequats based on ecofriendly caprylic acid were synthesized and their micellization behavior in water was explored systematically. The surface tension, wettability, foamability and stability, and measurements of melting and crystallization temperatures were employed to characterize the compounds. The functionalization of amidequats containing an alkoxymethyl substituent significantly enhances micellization properties compared to the structurally analogous anionic surfactant, sodium caprylate. Moreover, for optimized molecule geometries, electrical dipole moments were determined and correlated with surface activity. Experimental and theoretical studies indicate that amidequats with 12 carbon atoms in the alkyl chain exhibit the highest surface and foam-forming activity, making them promising emulsifiers. Due to this fact, oil-in-water (o/w) or water-in-oil (w/o) emulsions using the mentioned amidequat were prepared, and their rheological analysis, ζ potential, topography analysis, and particle size were studied. A detailed investigation of the surface properties of amidequats was undertaken to assess their potential application in the cleaning agent and cosmetic industries.

## Introduction

The development of surface-active ionic liquids (SAILs) is driven by the growing emphasis on safe, sustainable, and environmentally friendly chemistry.^1−4^ Therefore, it is crucial to synthesize SAILs, compounds that align with these principles, to effectively replace conventional surfactants or to serve as emulsifiers.^5,6^ SAILs share many properties with surfactants, including the ability to reduce surface and interfacial tension, to improve wettability, and to create stable, durable emulsions or foams.^2,7^ However, while SAILs can exhibit surfactant-like behavior, we believe equating the two groups of compounds is not entirely accurate.^8^ Like any ionic liquid, SAILs have melting points of less than 100 °C.^1^ Their “designability”, the ability to synthesize various cation–anion combinations, is a key property, enabling a wide range of industrial applications.^1,9,10^ In our recent works, we extensively discussed SAILs and their properties resulting from their amphiphilic (hydrophilic–hydrophobic) structure,^8,11,12^ specifically, compounds that had an amide group (amidequats) or imidazolium based on SAILs with an alkoxymethyl substituent.^8,11−13^ The functionalization of SAILs by introducing specific functional groups has attracted significant interest. According to the literature, compounds with hydroxyl, ether, ester, or amide functional groups are the most frequently described.^14−16^ Chauhan et al.^16^ suggested that the presence of an ester or amide group leads to a lower critical micelle concentration (CMC) and higher surface activity compared to conventional ionic surfactants or nonfunctionalized SAILs. They analyzed 3-alkyloxycarbonylmethyl-1-methylimidazolium and 1-alkyloxycarbonylmethylpyridinium SAILs and their nonfunctionalized analogues. Similarly, Wang et al.^17^ reached the same conclusion regarding amphiphilic imidazolium ILs with a carboxymethyl substituent.

The functionalization of SAILs is also environmentally beneficial: reducing their toxicity by appending ether or polyether side chains or enhancing antimicrobial activity in series of ester-functionalized imidazolium- and pyridinium-based amphiphilic compounds.^18^ In contrast, amide-functionalized SAILs can alter the behavior of micelles in aqueous solution due to elongation of the alkyl chain length or changes in van der Waals forces.^15^ The positive effect of introducing an amide group has been described in publications involving pyridinium, morpholinium, imidazolium, and ammonium cationic headgroups.^8,11,12,15,19^ All of the properties described above, along with the method of enhancing them by functional group modification, contribute to one goal: improving the effectiveness and applicability of SAILs. Both of these aspects are critical for the practical use of SAIL-based emulsions,^5,6^ especially in potential cream formulations where surface activity and micellization are key for creating stable and durable formulations.^20,21^ According to the literature, amphiphilic compounds participate in the formation of simple oil-in-water (o/w) or water-in-oil (w/o) emulsions or more complex multiple emulsions.^22^ Despite the popularity of “surfactant-free” Pickering emulsions,^23^ which have been studied since the early 20th century, new solutions are still being sought.^24^ However, the lack of surfactants in such systems often necessitates the introduction of products that do not irritate the skin.^25^ Therefore, using amphiphilic compounds like SAILs, especially those based on natural ingredients such as caprylic acid, seems to be a promising solution.^26−29^ Caprylic acid is a naturally occurring compound found in the milk of mammals such as goats and rabbits.^12^

In this work, we present newly synthesized surface-active amidequats with an alkoxymethyl substituent, focusing on their potential as emulsifiers and as replacements for conventional surfactants. To achieve this, a series of studies were conducted, examining the compounds’ ability to form micelles in aqueous solutions and interact with hydrophobic surfaces. The foamability of these surface-active amidequats was also evaluated as part of the research. The results were correlated with mathematical calculations to provide a comprehensive answer to the following question: How strongly do the elongation and positioning of the hydrophobic chain in the amphiphilic structure of the synthesized compounds affect their surface activity? In search of an answer, the most promising compound was selected and used as an emulsifier to create emulsions (o/w and w/o), which were characterized using microscopy, rheology, topography analysis, and drop size profiling. Understanding the properties of emulsions is essential for predicting product stability and ensuring that it meets consumer expectations, including aspects such as appearance, consistency, texture, and ease of use. To the best of our knowledge, surface-active amidequats with an alkoxymethyl substituent have not yet been studied for this purpose. Thus, we believe that achieving our research goals will broaden the industrial applications of these synthesized compounds and help bridge the gap in the utilization of SAILs.

## Materials and Methods

The entire experimental section is included in the Supporting Information (SI).

## Results and Discussion

### Synthesis and Characterization of Amidequats with an Alkoxymethyl Substituent

**Figure 1:**
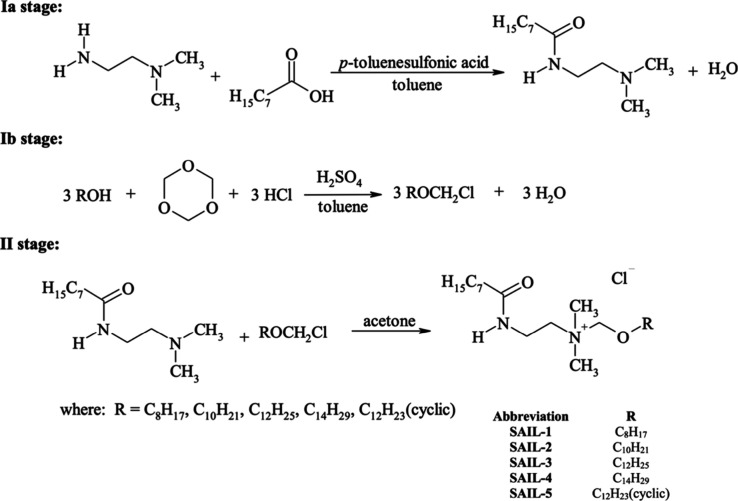
Synthesis of amidequats with alkoxymethyl substituents.

In order to obtain alkoxymethyldimethyl-*N*-[(2-octanamide)ethyl]ammonium chlorides (amidequats with an alkoxymethyl substituent), it was necessary first to synthesize an aminoamide and a quaternizing agent, specifically a chloromethylalkyl ether, and then, in the second stage, to perform the quaternization reaction of the aminoamide with this ether. Figure 1 shows the course of the performed syntheses. Stage Ia involved the preparation of compound *N*-[(2-dimethylamino)ethyl]octanamide. We had previously synthesized this compound using a different method involving an acid chloride.^12^ In this case, we opted for a more user-friendly synthesis method, replacing the troublesome octanoyl chloride with caprylic acid. A catalyst, *p*-toluenesulfonic acid, was necessary. To shift the reaction equilibrium toward the products, it was essential to continuously remove the water that collected in the Dean–Stark trap. The end of the reaction was easy to observe, as the water level ceased to increase. After the reaction was completed, the catalyst was removed, the solvent (toluene) was evaporated, and the product was purified by vacuum distillation. The distillate was collected at 170–172 °C under a pressure of 9 hPa, yielding the aminoamide with 84% efficiency. Simultaneously, during stage Ib, five quaternizing agents were synthesized: four linear chloromethylalkyl ethers containing an even number of carbon atoms in alkyl chain R (ranging from 8 to 14) and one cyclic ether containing 12 carbon atoms in the R substituent.

Chloromethylalkyl ethers were obtained by the chloromethylation of the corresponding alcohols using formaldehyde and gaseous hydrogen chloride. Formaldehyde was used in the form of 1,3,5-trioxane, a solid that was previously dispersed in liquid alcohol (octan-1-ol, decan-1-ol) or in a solution of alcohol in toluene (for solid alcohols, dodecan-1-ol, tetradecan-1-ol, and cyclododecanol). In turn, gaseous hydrogen chloride was produced due to the heat generated by mixing two concentrated acids (sulfuric acid(VI) and hydrochloric acid). It was crucial to maintain the reaction temperature in the range of 15–20 °C because, at temperatures below 15 °C, the reaction slows or even stops, while at temperatures above 20 °C, the selectivity decreases due to the competitive formation of formaldehyde dialkyl acetal. The chloromethylalkyl ethers were separated from water after the reaction and finally purified by vacuum distillation. The reaction yields and boiling points of the ethers under reduced pressure are presented in Table S1.

In the second stage of the reaction (Figure 1), amidequats with an alkoxymethyl substituent were obtained through the quaternization of aminoamide with chloromethylalkyl ether. The reactions had to be carried out under anhydrous conditions because the ethers are hydrolyzed in the presence of water, releasing alcohol, formaldehyde, and gaseous hydrogen chloride. The hydrogen chloride formed in the reaction can react with the aminoamide to form a hydrochloride (protic salt), making it practically impossible to separate the quaternary salt from the protic salt. The cationic substance content (purity) was determined based on two-phase titration, following the methodology described in our previous work.^12^ The remaining percentage up to 100% is water, as these compounds are hygroscopic. The structural formulas of the compounds were confirmed by NMR spectra, which are available in the Supporting Information. All obtained amidequats turned out to be ILs because their melting points did not exceed 100 °C. Table S2 presents the yields and purities of the obtained amidequats.

### Different Scanning Calorimetry (DSC) studies

**Figure 2:**
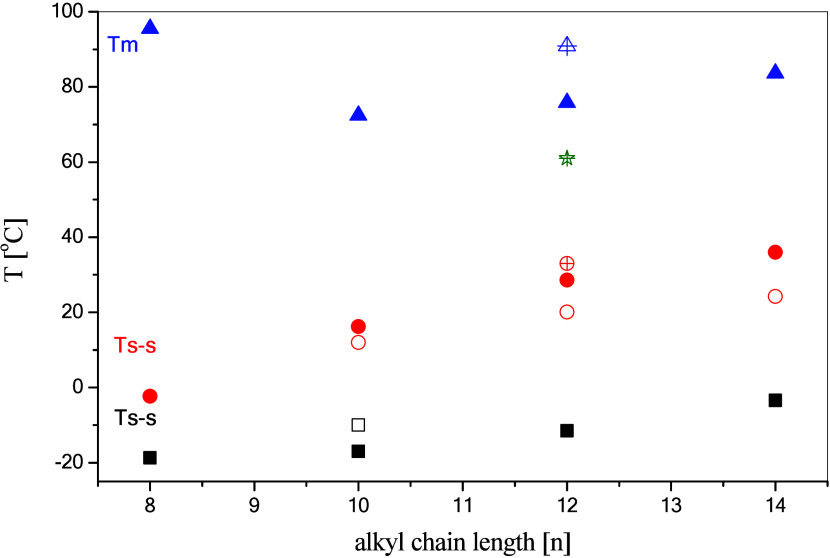
Relationship between the temperature of phase transitions during heating and alkyl substituent chain length. Additionally, points for **SAIL-5** were added (empty with a cross).

Table 1 presents the results obtained by the DSC method. As one can see, the synthesized compounds are characterized by the occurrence of a melting point (*T*_m_), several solid–solid transitions (*T*_s–s_), and crystallization temperatures (*T*_c_), which indicates that obtained amidequats are polymorphic compounds. These kinds of materials exist in more than one crystal form, which refers to various packing variations of the molecules connected with conformational disorder. In ionic liquids such as those tested in this work, with a cation having long alkyl substituents, disorder can be associated with availability of a range of conformations in the alkyl side chains (they have many rotational degrees of freedom).^30^ Changes in alkyl chain conformations can lead to changes in the density of the solid below the melting point.^31^ A solid–solid transition can also result from different arrangements of anions and cations, as well as changes in intermolecular interactions, such as hydrogen bonds; in the tested SAIL salts, amide groups capable of forming such bonds are present.^32^ Thus, each of the endothermic peaks recorded in the DSC thermogram during heating of the sample below the melting point corresponds to the solid–solid transitions (*T*_s–s_), “melting” of a different crystalline form of the same compound (see Figure S.7). Some solid–solid phase transitions manifest as two peaks, varying in their degree of separation. A clear trend is observed wherein, for SAIL with cations possessing shorter alkyl substituents, these transformations appear as a shoulder, while an increase in alkyl chain length results in greater peak separation. An additional graph (Figure 2) was prepared to illustrate the relationship between transformation temperatures and chain length (excluding **SAIL-5** containing a cyclic substituent). In general, melting temperatures decrease with an increase in alkyl chain length; however, **SAIL-1** exhibits a slightly lower melting temperature than **SAIL-2**, deviating from this trend. Additionally, the difference between the melting and crystallization temperatures is quite large, about 45–60 °C, but it is typical behavior for ionic liquids.^33^ This behavior indicates strong supercooling, and this delay in crystallization may be related to the kinetic barriers of nucleation (free energy barrier associated with the creation of the solid phase nuclei).^34,35^ Moreover, the synthesized compounds have quite high melting temperatures (maximum of peak), with the highest values for the tested compounds being in the range of 70–100 °C. However, the melting temperatures of the obtained compounds are below 100 °C (with the exception of **SAIL-2**, which exhibits a melting point around 100 °C; however, the onset of this transition occurs at 87 °C, allowing it to be classified as an ionic liquid), so all of the obtained compounds can be classified as ionic liquids.

**Table 1:** Melting (*T*_m_), Crystallization (*T*_c_), Solid–Solid Transition (*T*_s–s_), and Recrystallization (cold crystallization, *T*_cc_) Temperatures of Synthesized Compounds^a^

abbreviation	R	*T*_s–s_ (°C)		*T*_m_ (°C)	*T*_cc_ (°C)	*T*_c_ (°C)			
**SAIL-1**	C_8_H_17_	–18.3	–1.1	97.1		46.8	–23.1	–30.7	
**SAIL-2**	C_10_H_21_		4.6/15.6	100.9	0.83	54.8	–4.1	–11.8	
**SAIL-3**	C_12_H_25_	–6.6	21.5/28.3	87.9		23.7	9.3	–6.0	–30.0
**SAIL-4**	C_14_H_29_	–3.4	24.2/36.0	83.6		26.2	20.4	15.3	–33.6
**SAIL-5**	C_12_H_23_ (cyclic)	33.0	61.0	90.9	12.3	45.0	37.4	23.4	
**[Cap][Na]**		96.7	116.6	245		238.1	65.6		

a R = substituent. **[Cap][Na]** is an abbreviation for sodium caprylate.

### Surface Properties

**Figure 3:**
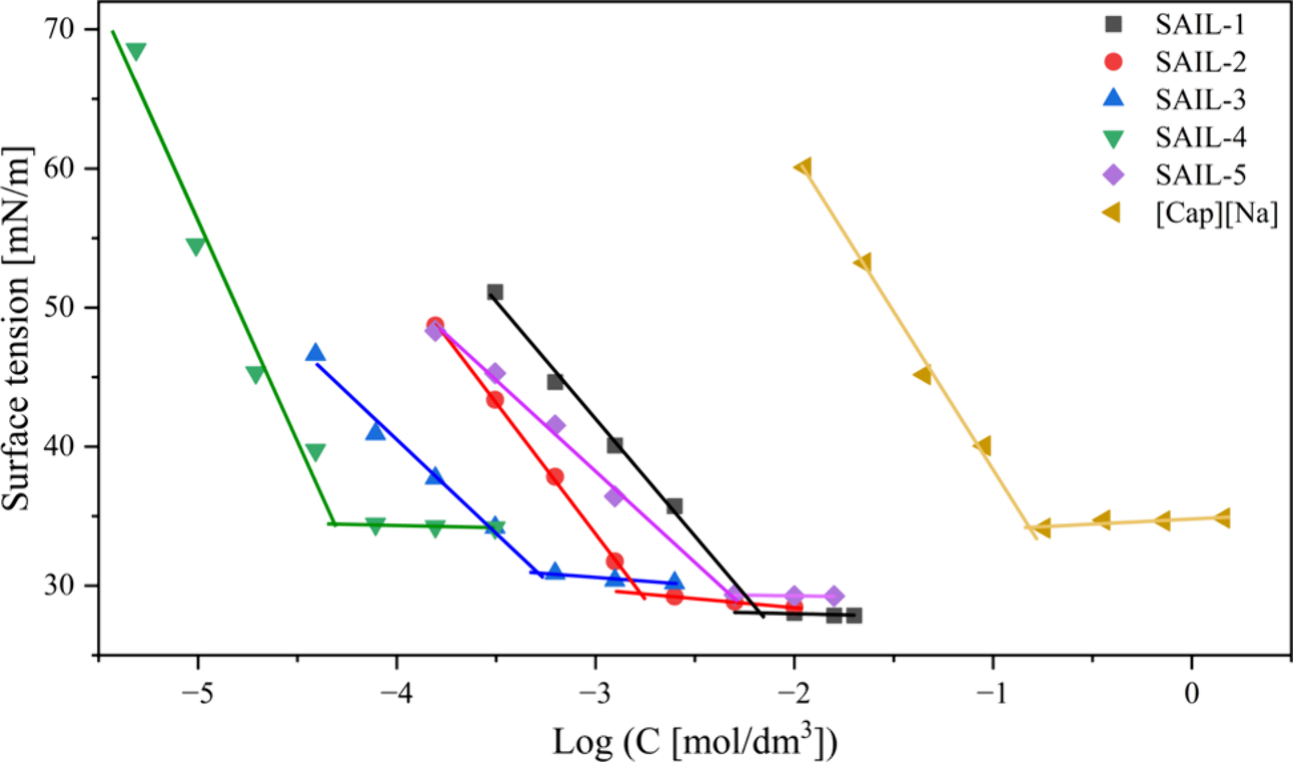
Relationship between surface tension and log of the concentration of compounds.

Compounds that can behave as surfactants demonstrate an inclination to reduce the surface tension of aqueous solutions. The surface tension of water gradually decreases with an increase in the concentration of these compounds until it levels off, which is associated with the presence of a plateau effect.^12^ The mentioned description is effectively illustrated by depicting the correlation between surface tension and the logarithm of compound concentration (log *C*), as presented in Figure 3.

Based on the graph presented above, the values for various parameters were determined, enabling the preparation of Table 2.

**Table 2:** Surface Properties of Synthesized **SAIL1**–**5** and **[Cap][Na]**^a^

abbreviation	R	CMC (mmol/L)	γ_CMC_ (mN/m)	Π_CMC_ (mN/m)	pC_20_	Γ_max_ (×10^6^ mol/m^2^)	*A*_min_ (×10^19^ m^2^)	Δ*G*_ads_° (kJ/mol)	CA (deg)
**SAIL-1**	C_8_H_17_	6.72 ± 0.92	28.0 ± 0.5	44.8 ± 0.1	3.64 ± 0.52	3.63 ± 0.72	4.57 ± 0.72	–25.16 ± 0.32	47.49 ± 2.42
**SAIL-2**	C_10_H_21_	1.70 ± 0.34	29.4 ± 0.2	43.4 ± 0.8	4.01 ± 0.59	2.88 ± 0.56	5.76 ± 0.72	–30.0 ± 0.65	42.13 ± 2.92
**SAIL-3**	C_12_H_25_	0.51 ± 0.42	30.9 ± 0.3	41.9 ± 0.5	4.91 ± 0.21	2.27 ± 0.52	7.32 ± 0.65	–36.66 ± 0.83	56.03 ± 2.64
**SAIL-4**	C_14_H_29_	0.05 ± 0.09	34.5 ± 0.1	38.3 ± 0.5	4.88 ± 0.39	7.94 ± 0.21	2.09 ± 0.72	–28.68 ± 0.82	87.36 ± 1.03
**SAIL-5**	C_12_H_23_ (cyclic)	4.72 ± 1.12	29.4 ± 0.6	43.4 ± 0.1	4.11 ± 0.51	2.77 ± 0.56	5.98 ± 0.53	–29.67 ± 0.99	53.77 ± 3.02
**[Cap][Na]**		153.93 ± 1.23	34.2 ± 0.9	38.6 ± 0.2	1.64 ± 0.42	4.51 ± 0.87	3.68 ± 0.72	–12.95 ± 0.21	89.95 ± 1.32

a R, substituent. **[Cap][Na]** is an abbreviation for sodium caprylate.

Through the analysis of the surface activity results obtained, crucial aspects related to the structure of the synthesized compounds will be specifically examined. The first pertains to the elongation of the alkyl chain, while the second focuses on the impact of the presence of an alkoxymethyl substituent. Still another issue is associated with the arrangement of the alkyl substituent.

The tested amphiphilic compounds effectively reduce the surface tension (γ) of water, as evidenced by the surface tension at the CMC (γ_CMC_) parameter values obtained. Interestingly, γ_CMC_ values were increasing with alkyl chain elongation. The lowest value was obtained for **SAIL-1** (28.0 mN/m), and the highest for **SAIL-4** (34.5 mN/m). Moreover, in the case of **SAIL-5**, the γ_CMC_ value is lower than that of **SAIL-3**. This is possible because of the cyclic structure formed by the alkyl chain. γ_CMC_ is coherently connected with γ_CMC_. The values of surface pressure at the CMC (Π_CMC_) differ only slightly for the shorter alkyl chain homologues form C_8_ to C_12_. However, for **SAIL-4**, the effectiveness of the surface tension reduction is lower than for the mentioned compounds.

In order to analyze the surface activity of the compounds, a very important indicator, CMC, was used. Therefore, we will try to interpret the obtained values of this parameter and, in the next section, correlate them with the results describing the propensity of SAILs to form stable foam. CMC values for the tested compounds range from to 6.72 to 0.05 mmol/L and decrease with an increase in alkyl chain length. However, if one compares the CMC values of **SAIL-3** and **SAIL-5**, a very interesting relationship is observed. It has been shown that when amphiphilic compounds are made of straight alkyl chains, the propensity for micellization increases, as evidenced by lower CMC values.^36^ On the other hand, if in the molecule of the alkyl chain adopts a closed structure, i.e., cyclic, then the surface activity of the compound decreases, and the evidence is similar CMC values as for SAIL with a much shorter chain length.^19^ Another very important point is based on the number of alkyl chains. For the analyzed **SAIL1–5**, the CMC value is at least 23 times lower than that for **[Cap][Na]**, a compound containing only one alkyl chain. Therefore, it can be summarized that the more asymmetric the molecule is, the higher the surface activity the compound shows. Comparing the efficiency of micellization and CMC values of amide-functionalized **SAIL1–5** to the series of conventional surfactants, it is clear that the introduction of an amide group affects the formation of micelles in aqueous environments. According to Garcia et al.,^14^ the CONH- group of compounds is involved in the formation of intramolecular hydrogen bonds between neighboring molecules (water and surface-active amidequats). On the other hand, the second reason for the increased surface activity of SAILs may be due to the presence of an alkoxymethyl substituent, and in fact, it is oxygen that may also participate in the intermolecular bonds formed, as inferred above by Garcia et al.^14^ Also, Kapitanov et al.^37^ suggested that the polarity for amide groups in the side chain is higher than if analyzed in the context of alkyl chains. As previously mentioned, the existence of amide-functionalized groups has a notable influence on micellization and a similar effect on adsorption at the air/water interface.^15^ On the other hand, when comparing the CMC values of our synthesized compounds with literature data for the conventional surfactant, didecyldimethylammonium chloride (DDAC) with a CMC value of ∼2 mM,^38^ it is evident that only **SAIL-1** and **SAIL-5** exhibit lower surface activity. This can be attributed to the lower degree of hydrophobicity of the tested compounds. Whereas comparing the ability to create micelles of **SAIL1–5** with another series of cationic surfactants, namely, alkyltrimethylammonium bromides (**C**_**10**_**TAB**, decyltrimethylammonium bromide; **C**_**12**_**TAB**, dodecyltrimethylammonium bromide; **C**_**14**_**TAB**, trimethyltetradecylammonium bromide; **C**_**16**_**TAB**, hexadecyltrimethylammonium bromide) for which the CMC values are 67.00, 15.00, 4.08, and ∼1 mM,^39−41^ respectively, it can be emphasized that surfactants used in industry, despite having one long alkyl chain, lead to harder micellization.

Generally, the values of adsorption efficiency (pC_20_) increased with the number of carbon atoms in the hydrophobic chain (**SAIL-4** is an exception). In the literature, this is a well-known tendency that is directly related to the free energy resulting from the adsorption of the methylene group at these interfaces. Moreover, according to one of the most important theories, as the length increases, the difference between the values of the pC_20_ is about 0.60. In contrast, the difference is much higher for the synthesized ones, which may indicate that the surface concentration, which is nearly the value of saturation, can be achieved with more than 50% of the bulk phase of the compound concentration.^12,42^

As reported in Table 2, the highest Γ_max_ accompanied by the lowest *A*_min_ value, and vice versa, was observed. This relationship defined the packing of the molecules of a compound at the air–solution interface. The lowest *A*_min_ value obtained for **SAIL-4** suggests that for this compound, the compound ions were closely packed. Moreover, it indicates a possible so-called shielding effect or could be a consequence of intermolecular and intramolecular H-bonding of the amide-functionalized moiety.^43^ The values of Δ*G*_ads_° are negative, which indicates the spontaneous course of the adsorption.^44,45^ Additionally, it was noted that an increase in the hydrophobicity of compounds correlates with Δ*G*_ads_°.^46^ Some researchers have also sought to establish connections between Δ*G*_ads_° and the structural characteristics of amphiphilic compounds.^47−49^ For example, the Δ*G*_ads_° for the cationic surfactant dodecylethyldimethylammonium bromide (DAB) is −23.7 kJ/mol,^5^ whereas for non-ionic surfactant 2-[4-(2,4,4-trimethylpentan-2-yl)phenoxy]ethanol (Triton X-100), the Δ*G*_ads_° is −46.2 kJ/mol.^50^ These findings indicate that fatty alcohol ethoxylates exhibit more negative adsorption energy compared to cationic amphiphilic compounds, such as the synthesized compounds studied here.

In summary, based on the discussion described above, one can see how a key role in the case of cationic surfactants (including amidequats) is played by the headgroup, its size, and the hydrophobic groups attached to it.^1,51^ These factors are important for the adsorption of SAILs at the air–solution interface and micellization. However, we feel that in there are still some gaps in the proper interpretation of interfacial as well as in-solution phenomena and the strict role of the hydrophilic–hydrophobic structure of amphiphilic compound molecules.^42^

On the other hand, the values of CA are within the limit indicating partial wetting of the model surface, which is consistent with the literature data.^52−54^ Despite the fact that the best wettability was shown by **SAIL-2** with 10 carbon atoms in the substituent, there is still a chance to observe the relation that as the length of the alkyl chain increases, the wettability decreases. Interestingly, the highest CA value was obtained for **[Cap][Na]**, which is comparable to **SAIL-4**. Undoubtedly, this is a rather unique observation given that **[Cap][Na]** has only one long alkyl substituent in the structure while **SAIL-4** has two.

### Effect of Hydrophobic Chain Elongation on Physicochemical Properties

The degree of wetting and contact angle are determined by the balance of the liquid’s cohesion and adhesion to the material’s surface. Wetting is minimized when cohesion predominates and increases when adhesion dominates. The contact angle is a quantitative indicator of wettability inversely related to it. A large angle indicates the poor wettability of the solution, which means that the droplet does not spread significantly on the surface.^55^ A value of CA of less than 90° (low CA value) typically suggests that the surface is very wettable by the liquids. Low-energy surfaces interact with liquids primarily via van der Waals forces, typically described as a combination of interactions between induced dipoles, permanent dipoles and induced dipoles, and permanent molecular dipoles.

The permanent dipole moments of the compounds investigated in aqueous solutions were estimated using a quantum mechanical modeling method. The first step was to look for molecular geometry in the energy minimum. The potential energies of the molecules’ rotamers were analyzed for this purpose. Rotations of molecular fragments have been performed around the C–C bond (dihedral angle α, Figure S.8a) and the C–O bond (dihedral angle β, Figure S.8b) for all systems that were investigated.

**Figure 4:**
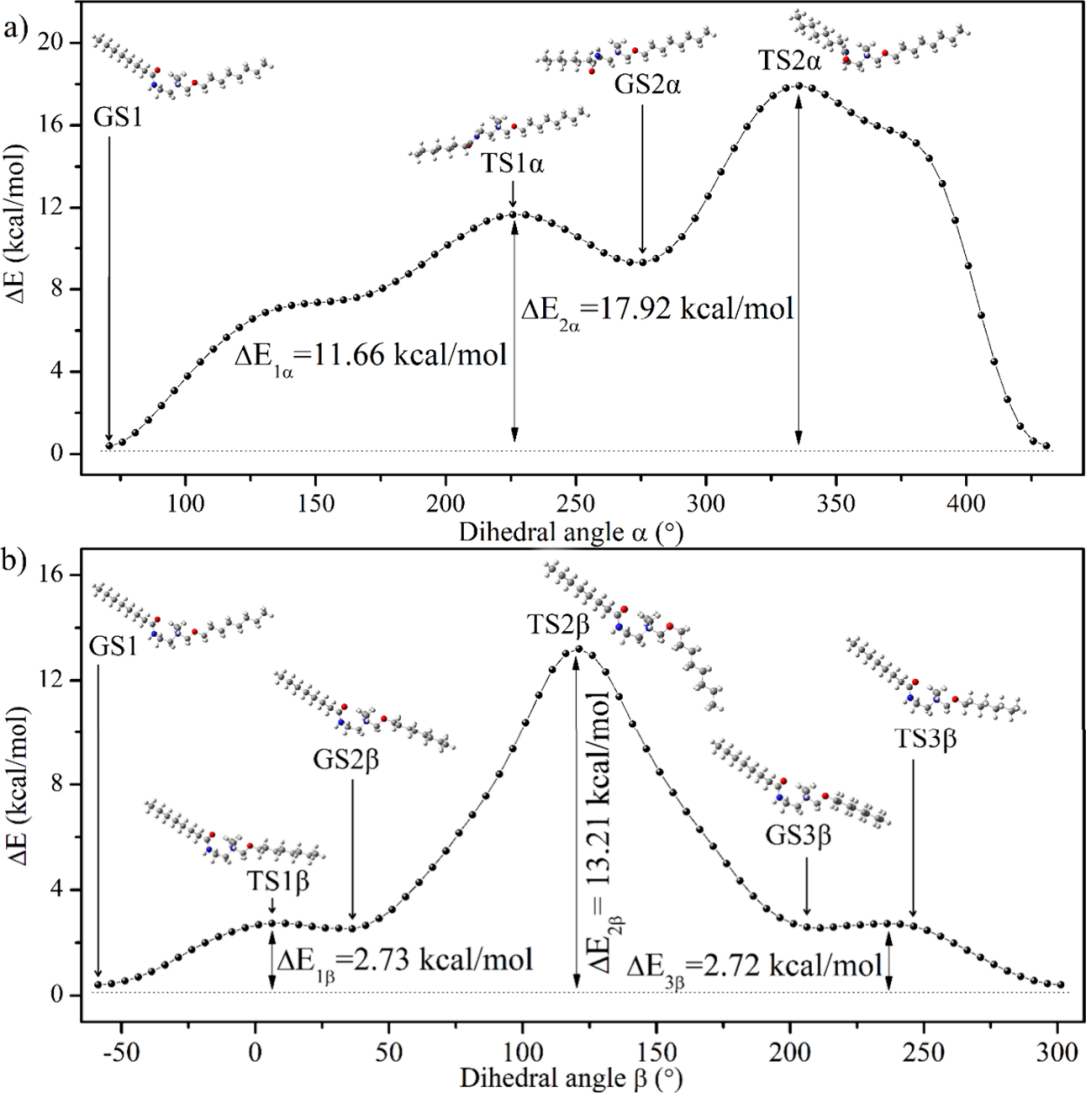
Scans of the total energy as a function of rotation around the (a) C–C bond (dihedral angle α) and (b) C–O bond (dihedral angle β) for **SAIL-1**. The calculations were done using the DFT methods cam-B3LYP/6-311++G(d,p). The energy value has been normalized; the minimum energy for GS1 (*E* = −1086.79405453 hartree) equals zero. GS means the ground state, and TS a transient state.

Figure 4 displays scans of the total energy as a function of rotation around the C–C bond (dihedral angle α) (a) and C–O bond (dihedral angle β) for **SAIL-1**. The potential energy scan for several geometries reveals the existence of two minima, GS1 and GS2α when rotating around the C–C bond. In the figure, we observe transition states between the minima, denoted as TS1α and TS2α. Regarding the rotation around the C–O bond, we see three minima: GS1, GS2β, and GS3β. We find the transition states between the minima: TS1β, TS2β, and TS3β. The molecular geometries of **SAIL-1** are depicted in Figure 4, as well. The ground state (GS1) with the lowest energy (*E* = −1086.7940 hartree) is characterized by a bent molecule with a dihedral angle of 33.94° between the aliphatic chains. Table S3 summarizes the dihedral angles observed in the investigated compounds during rotations around the C–O bond. The data reveal that molecules with a more linear structure exhibit greater potential energy. Transition state TS2β corresponds to almost linear molecules with a dihedral angle ranging from 164° to 179°. The energy of the TS2β transition state is 13.21 kcal/mol higher than that of the GS1 state. The value of the molecule’s dipole moment is influenced by its geometry and, consequently, by the magnitude of the angle between its aliphatic chains. The molecule’s moment for the compounds under investigation increases with an increasing angle (Figure S.9).

Considering the energy of the states and the energy barriers between them, the most probable geometry, i.e., for which we observe an energy minimum, was selected for further study. The optimized geometries for all examined compounds in vacuum and aqueous solutions are shown in Figure S.10. As the length of the aliphatic chain increases and the dielectric constant of the medium varies, there is a modest variation in the angle between aliphatic chains, approximately 10°. Furthermore, when the length of the aliphatic chain increases, the electric dipole moment also increases. Figure S.10 illustrates that a solvent’s presence enhances the molecules’ electric dipole moment and alters its orientation.

**Figure 5:**
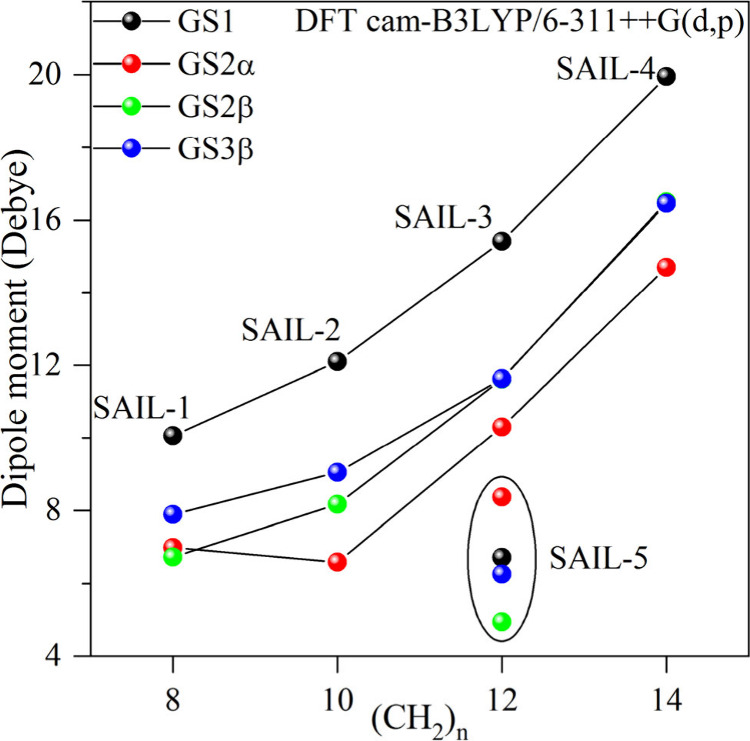
Dipole moments calculated for **SAIL1–5** in ground states. Calculations were performed at the cam-B3LYP/6-311++G(d,p) level of theory.

Figure 5 and Table S4 display the computed values of the electric dipole moment for the examined compounds throughout various states. The electric dipole moment for **SAIL1–4** is most prominent in the lowest-energy state. The compound with the lowest dipole moment among all those investigated is **SAIL-5**, in which the alkyl chain adopts a closed structure. An analysis was conducted to examine the impact of the calculation method on the values of the dipole moment (see Figure S.11). Table S5 compares the dipole moments of **SAIL1–5**. These dipole moments were estimated using the B3LYP, cam-B3LYP, and ωB97x-D functionals, along with the 6-311++G(d,p) and 6–31G(d,p) bases. The lowest dipole moment of 6.43 D (B3LYP) was achieved in a vacuum for **SAIL-5**, a compound in which the (CH_2_)_12_ group forms a cyclic ring. The dipole moments of compounds containing aliphatic groups with varying chain lengths range from 9.81 to 19.76 D.

**Figure 6:**
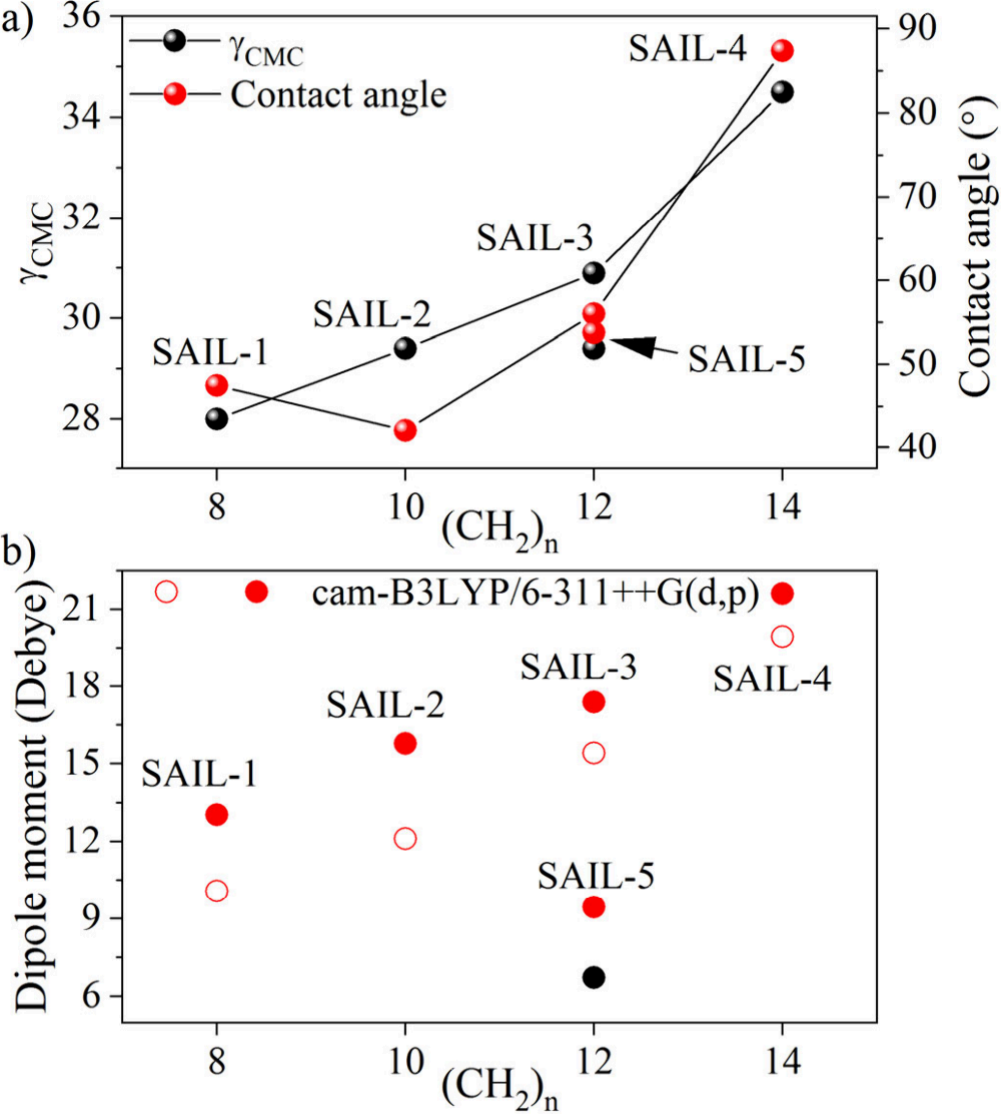
(a) Surface tension (γ_CMC_) and CA obtained for **SAIL1–5**. (b) Dipole moment in vacuum (empty symbols) and in solvent (filled symbols) calculated for **SAIL1–5** at the cam-B3LYP/6-311++G(d,p) level of theory.

Figure 6 illustrates the relationship between the quantity of the methylene group in the aliphatic chain and the values of γ_CMC_, CA, and dipole moment. It is demonstrated that the longer the aliphatic chain, the larger the ionic liquid’s contact angle and dipole moment. When the dipole moment of the molecules increases, cohesion prevails, and wettability decreases. **SAIL1–4** exhibited a nearly 2-fold increase in dipole moment and CA values when the aliphatic chain length (*n*) increased from 8 to 14. Aliphatic chains should have the minimum possible length or form cyclic structures to achieve better wettability.

### Foam Properties of SAILs

**Figure 7:**
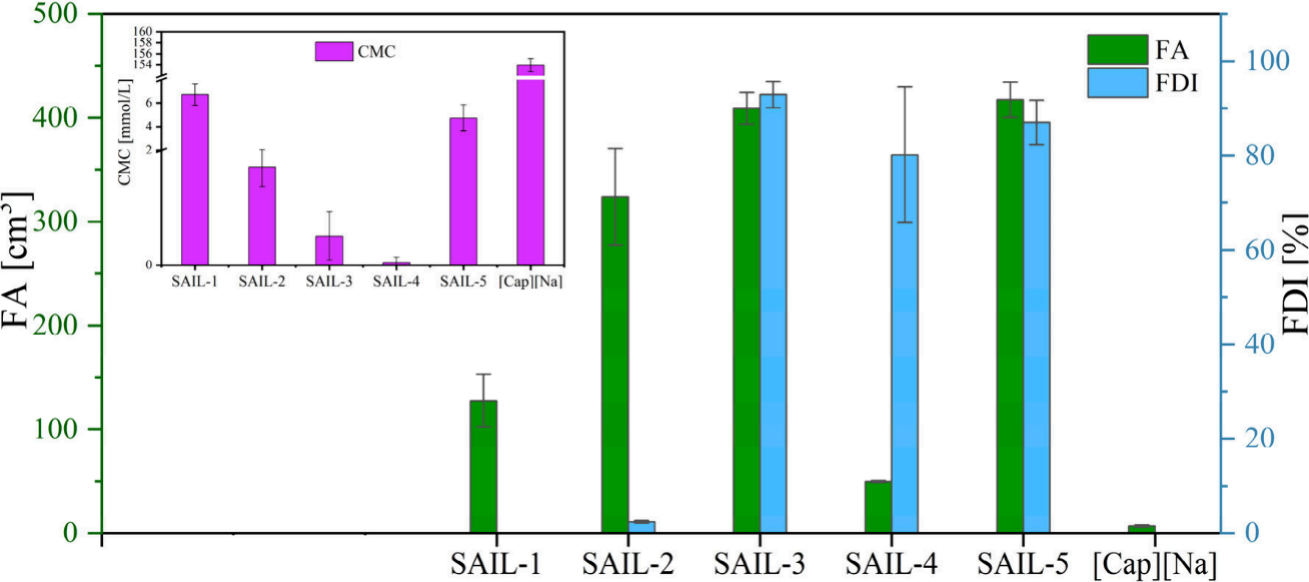
Foaming properties of **SAIL1–5** and **[Cap][Na]** based on FA and FDI values.

To confirm the use of the synthesized amidequats as potential replacements for commercial surfactants, their foaming properties were investigated. For this purpose, two indices, foamability (FA) and foam durability index (FDI), were determined, and their values were compared with the CMC values of the compounds (see Figure 7).

Looking at the results obtained, it is evident that the compounds exhibit significantly better foaming properties compared to the reference substance **([Cap][Na]**), highlighting the potential of synthesized **SAIL1–5**. Moreover, as the alkyl chain in the alkoxymethyl substituent of amidequats is extended, FA increases up to the C_12_H_25_OCH_2_- substituent, after which it decreases sharply. Interestingly, in our studies, the highest FA value was obtained for **SAIL-5**, while the highest FDI was achieved with **SAIL-3**. Therefore, we can conclude that the optimal alkyl chain length for efficient foaming agents is 12 carbon atoms. Notably, the foam properties of these two compounds are nearly identical, but their CMC values differ significantly. This discrepancy might be due to the lack of elasticity in the monolayer film of **SAIL-5**, whose alkyl chain forms a more rigid structure. Kim et al.^56^ described a general tendency: the lower the CMC value, the more efficient the compound as a foaming agent. However, foam formation typically begins near or above the CMC, where there are sufficient surfactant molecules at the air–water interface to reduce surface tension and stabilize gas bubbles. Below the CMC, foam is usually unstable and collapses quickly due to insufficient surfactant at the interface to stabilize the bubbles. Above the CMC, foam stability improves, as micelles act as reservoirs, replenishing surfactant molecules at the bubble surface and prolonging foam life. While this phenomenon likely holds true for the authors’ findings, it is not applicable in our study, as we focused exclusively on concentrations at the CMC of the compounds.

### AFM Analysis

In order to gain a deeper insight into the surface activity of the synthesized amidequats, a topographical analysis of the behavior of the compounds on a mica surface was performed. It is worth emphasizing that the analysis was conducted on two of all synthesized SAILs. This was done to investigate how compounds behave, in which the amphiphilic part has a simple alkyl chain (**SAIL-3**) and an alkyl chain that takes a cyclic form (**SAIL-5**).

**Figure 8:**
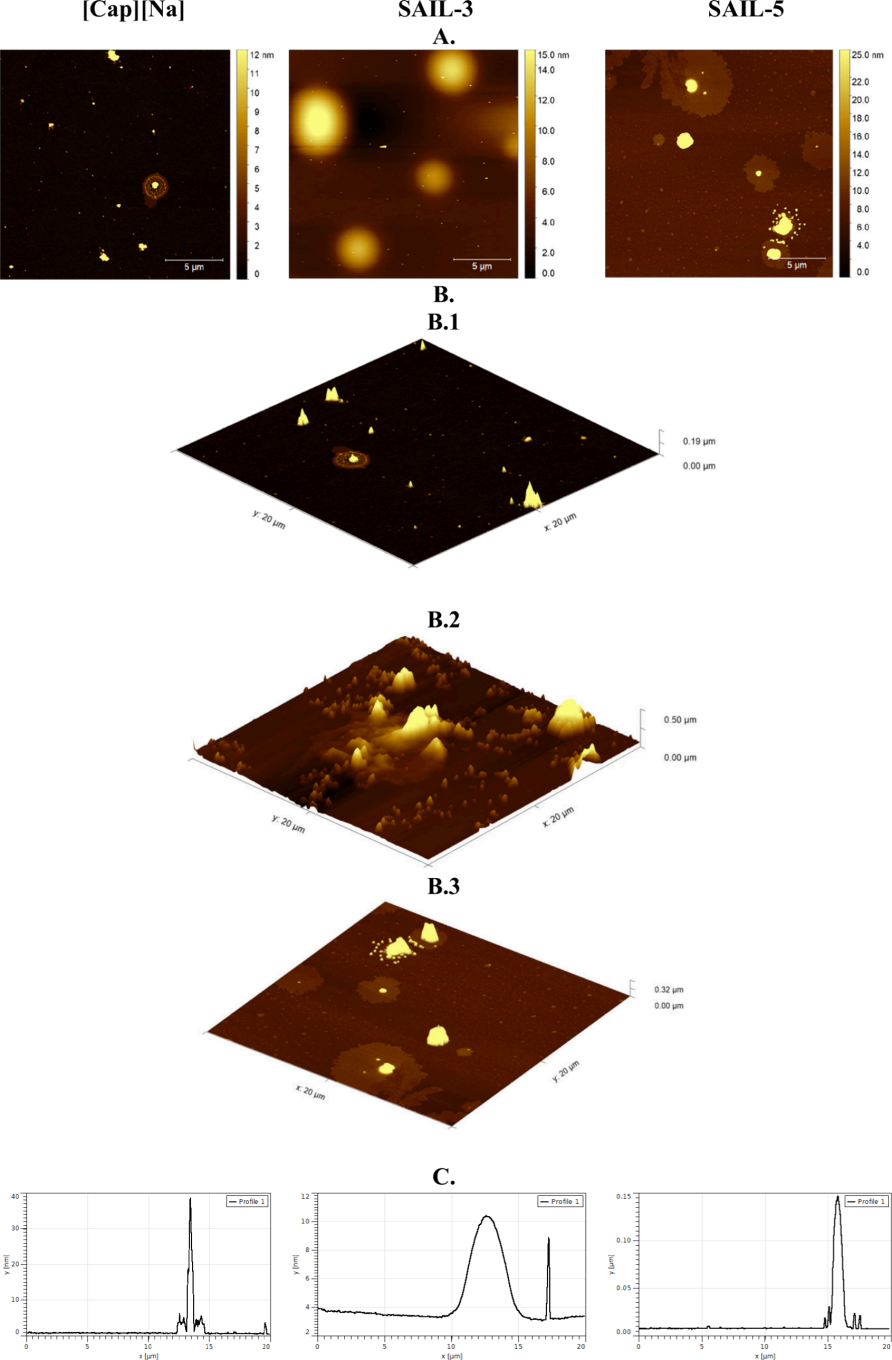
AFM results for SAILs showing the difference with surface coverage. (A) Topography of selected areas of compounds. (B.1–B.3) 3D view of the test surfaces of **[Cap][Na]**, **SAIL-3**, and **SAIL-5**, respectively. (C) Three profile curves for selected deposits.

Based on the results presented in Figure 8, one can observe the formation of aggregates. While in the case of **SAIL-3**, the aggregates formed can be stated to resemble spheres with a very low height (around 7 nm) but a relatively large diameter (2700 nm). In contrast, the objects formed by **SAIL-5** and **[Cap][Na]** are rough and varied in height, which does not directly reflect the spheres characteristic of surfactants. They are approximately 300 and 40 nm in height and 1000 and 500 nm in diameter, respectively.

In light of the data presented above and the discussion of the results (Surface Properties), it can be hypothesized that it is the **SAIL-3** compound that may be prospective at this stage as a surface-active agent.

### Analysis of Emulsions Based on SAILs

**Figure 9:**
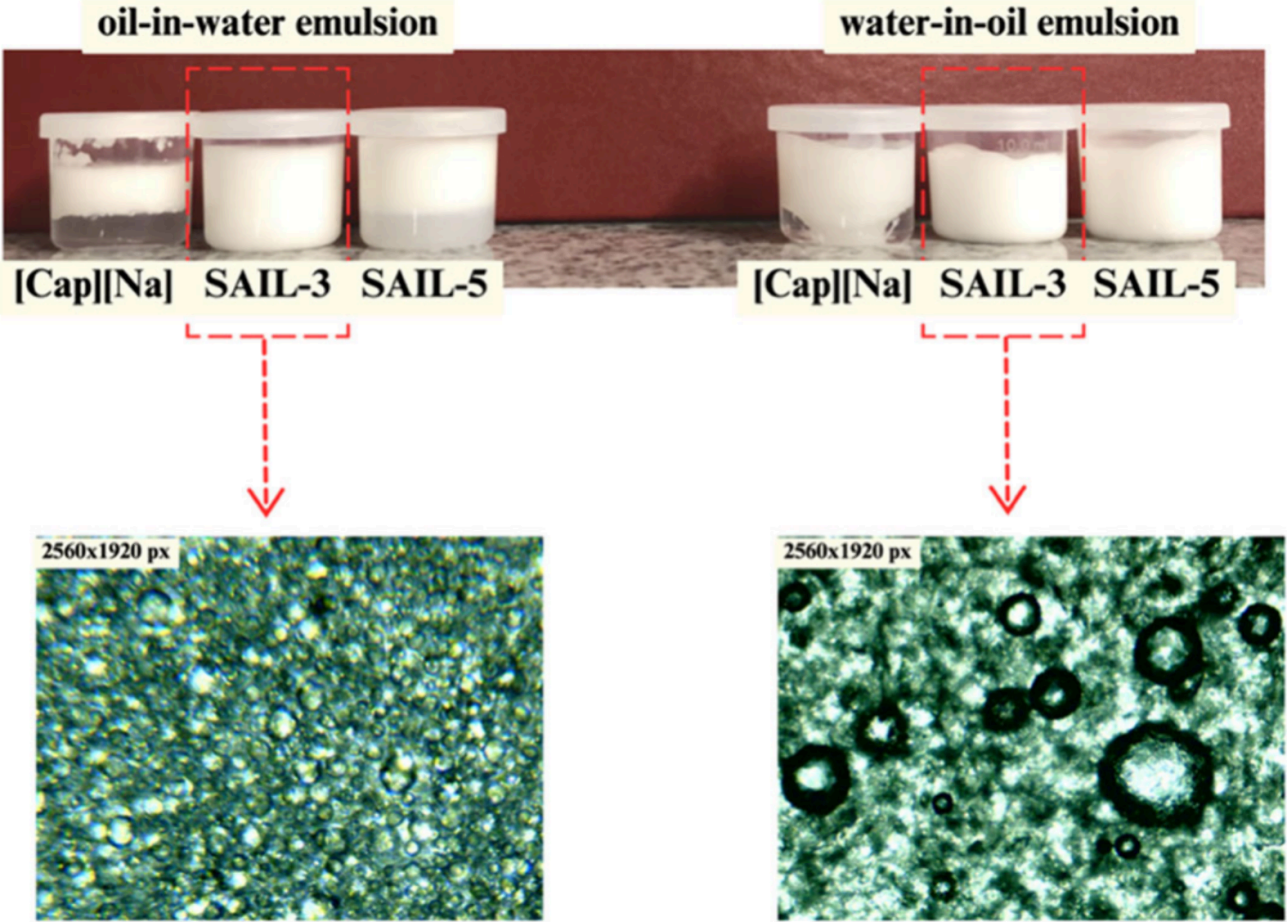
Graphical representation of the types of formation based on **SAIL-3**, **SAIL-5**, and **[Cap][Na]**.

According to the observation of the study, two compounds (**SAIL-3** and **SAIL-5**) were selected from among the synthesized SAILs, from which the ability to be an emulsifier was tested to consequently form a stable and durable emulsion (see Figure 9). The choice of compounds was dictated by high surface activity and the desire to test the influence of alkyl chain formation.

Based on the centrifugal tests carried out, differences in emulsion stability depending on the emulsifier used were demonstrated. Formulations that used **[Cap][Na]** and **SAIL-5** as emulsifiers became delaminated. Therefore, we decided to further characterize only the emulsions with **SAIL-3** prepared according to the literature.^57^

**Figure 10:**
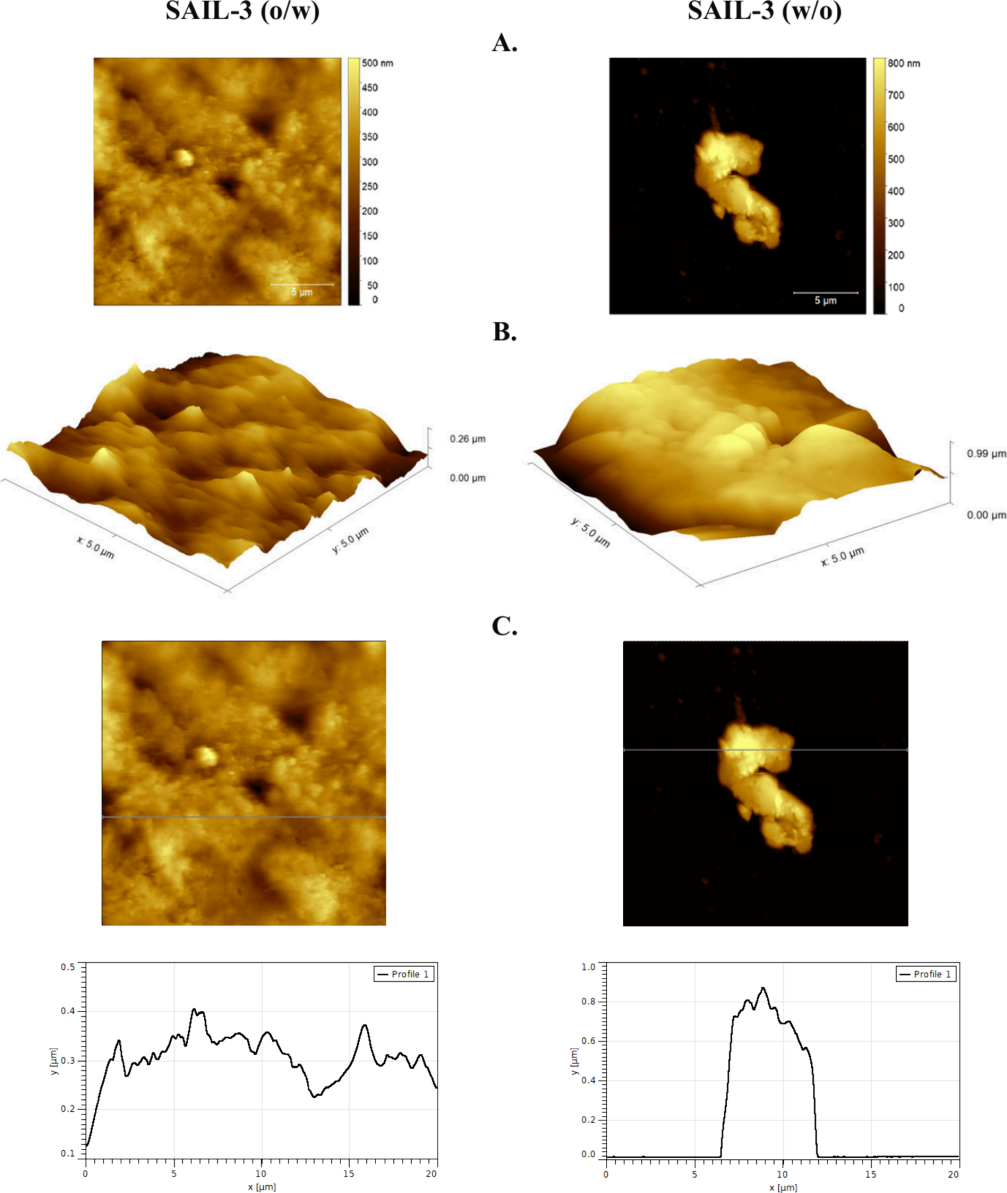
AFM results for SAILs showing the difference with surface coverage. (A) Topography of selected areas of emulsions. (B) 3D view of the test surfaces. (C) Profile curves for selected deposits.

**Figure 11:**
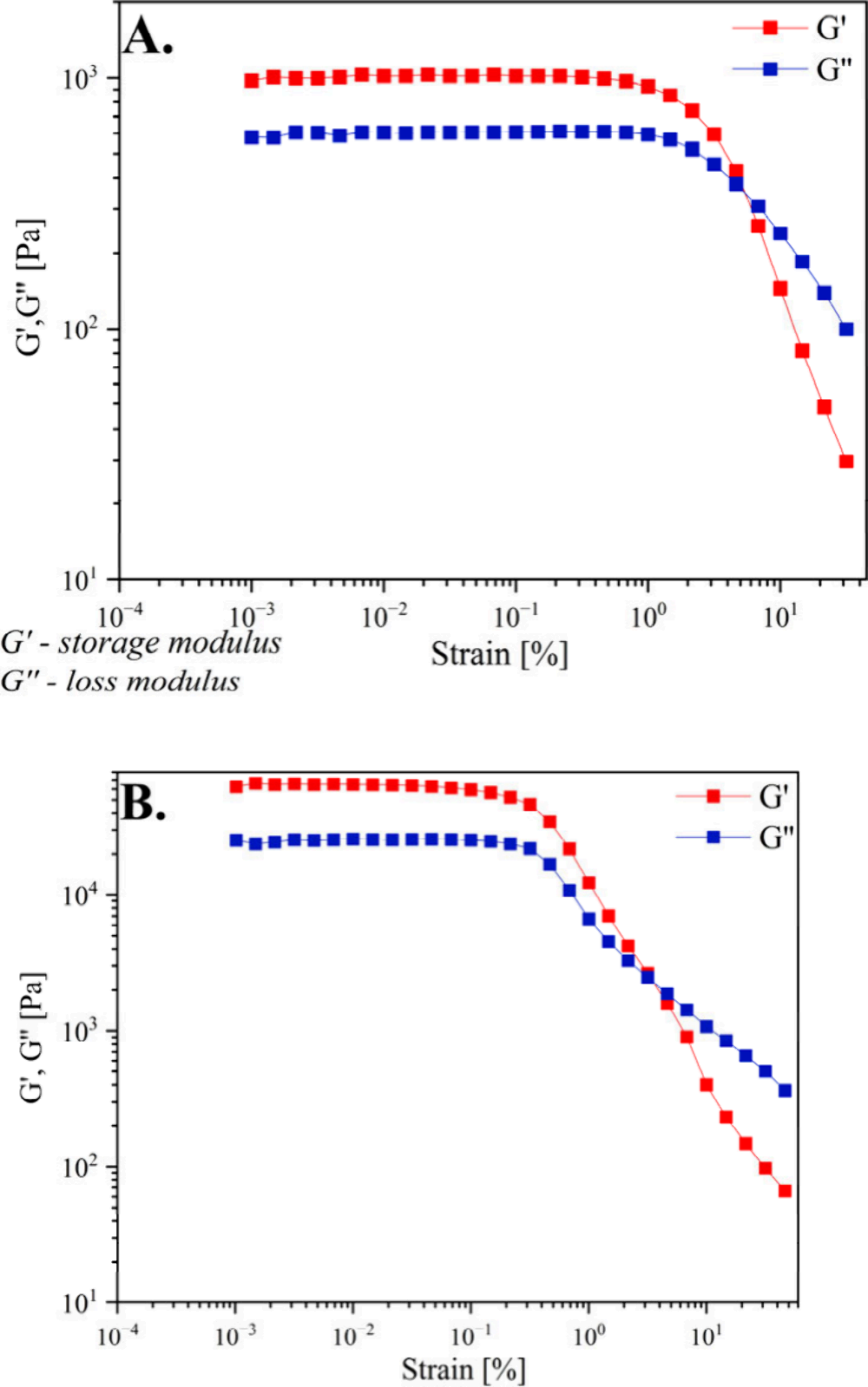
Examination of the dependence of the storage modulus (*G*′) and loss modulus (*G*″) on the strain amplitude (γ_0_) of (A) o/w and (B) w/o emulsions with **SAIL-3**.

**Figure 12:**
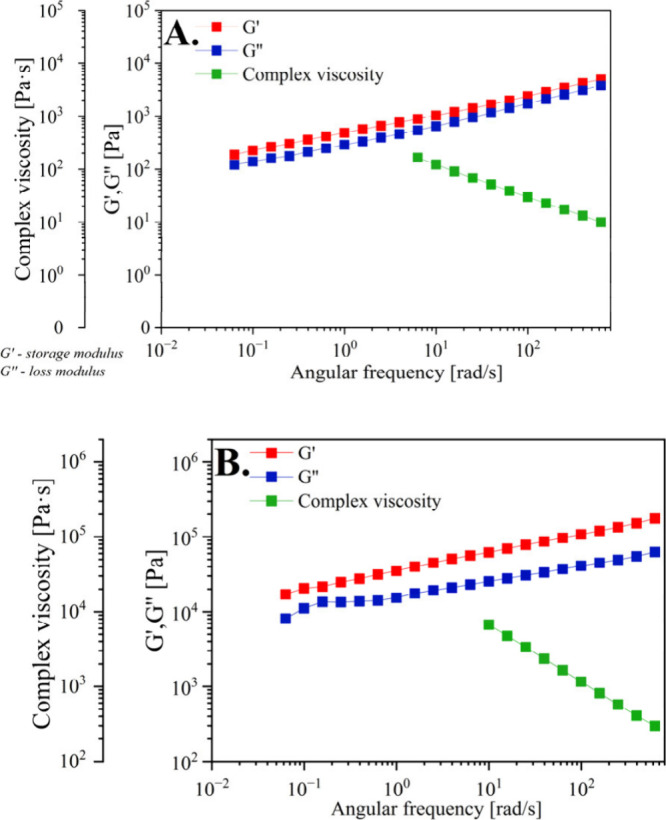
Frequency sweep tests of (A) o/w and (B) w/o emulsions with **SAIL-3**.

**Figure 13:**
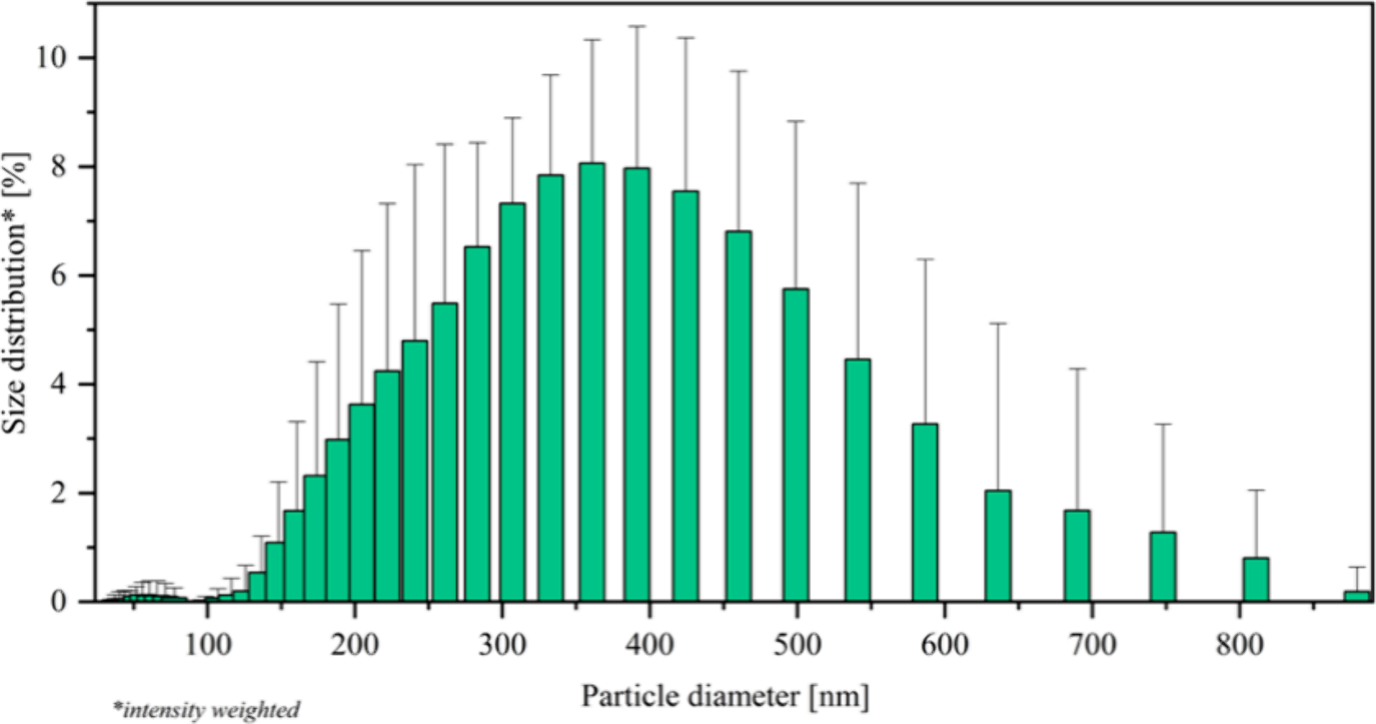
Size distribution of o/w emulsion droplets.

Rheological analysis, ζ potential, topography analysis, and the particle size of the emulsion were studied, and the results are graphically presented in Figures 10–13, respectively. Additionally, the density of the emulsions was investigated. For the o/w emulsion, the density was 0.8532 ± 0.050 g/mL, while for the w/o emulsion, it was 0.9021 ± 0.014 g/mL.

Considering the potential use of **SAIL-3** as an emulsifier, we decided to examine the surface coverage of model o/w and w/o emulsions. As shown in Figure 9, there are differences in the manner of surface coverage. Referring primarily to the work of Doković et al.,^58^ whose observed droplets had spherical or elliptical shapes, the surface coverage in the prepared o/w emulsion forms a layer with an irregular shape. The roughness average of the o/w emulsion is 1.78 nm, and the maximum height of the roughness is 15.56 nm; in the case the w/o emulsion, the roughness average is 3.61 nm and the maximum height of the roughness is 25.85 nm.

Figure 11 shows the results of the strain sweep test for w/o and o/w emulsions with **SAIL-3**, presenting the dependence of the storage modulus (*G*′) and loss modulus (*G*″) on strain amplitude (γ_0_). The storage modulus (*G*′) characterizes the elastic properties of the emulsions, while the loss modulus (*G*″) characterizes the viscous properties of the emulsions. For both emulsions, the range of linear viscoelasticity ends at a relatively low strain amplitude (γ_0_ values of 0.15% and 0.03% for o/w and w/o emulsions, respectively). The low strain amplitude values can be correlated with the images of emulsion droplets shown in the photographs (Figure 9).

The droplets of both emulsions are strongly flocculated, whereas the forces causing the droplets to aggregate are weak, resulting in the breakdown of the microstructure formed in the resting state at low strain amplitude values. The results of the frequency sweep tests presented in Figure 12 are also characteristic of flocculated emulsions. This experiment was conducted within the linear viscoelastic range at a strain amplitude γ_0_ of 0.01%. Throughout the entire frequency range, storage modulus *G*′ is greater than loss modulus *G*″, and the value of the loss tangent (tan δ = *G*″/*G*′, with tan δ → 0 for elastic bodies and tan δ → ∞ for Newtonian fluids) is approximately constant at around 0.65 for the o/w emulsion and around 0.4 for the w/o emulsion. The shape of the obtained mechanical spectra and the values of tan δ are characteristic of so-called “weak gels”.

To further investigate the stability of the produced emulsions, we decided to analyze the ζ potential (ZP), which represents the difference in electrical charge between the tightly bound layer of ions surrounding the micelle particles and the stationary fluid layer around them.^59^ In the literature, such stability tests are carried out on o/w emulsions.^59,60^ Kaszuba et al.^61^ summarized that particles with an absolute magnitude of ZP > 30 mV are usually considered to be stable. They explained their observation by the presence of a sufficiently strong electrical charge of droplets, which determines the repulsive forces are predominant in the emulsion systems. The suggestion mentioned above may be true, but ZP was significantly influenced by emulsifier type. Our emulsion with a cationic surface-active agent as an emulsifier demonstrates “good” stability, with ZP values oscillating at −47 ± 7 mV, which is like those of emulsions produced using non-ionic surfactant Tween 80 as an emulsifier (with an electrical charge of −35.8 ± 3.8 mV).^61^ Guerra-Rosas et al.^62^ reported the weakest electrical charge ranging from −6.53 to −15.20 mV. This is most likely due to the study of emulsions containing high-methoxyl pectin, Tween 80, and a variety of essential oils. Looking through the results obtained in this work and in the literature presented here, we can clearly summariz that the emulsifier’s amphiphilic nature can control the surface charge because surface-active molecules adsorb in an oriented manner at the o/w interface, leading to lower interfacial tension and as a consequence to more negatively charged particles. Another consideration relates to emulsification processes themselves, which can also have an impact on ZP values, such as obtaining emulsions by microfluidization. This issue will be addressed in our future deliberations.

The hydrodynamic size distribution of the suspended phase droplets predominantly falls within the range of 200–500 nm, further supporting the uniformity and stability of the emulsion (see Figure 13). From these results, we can confirm that the micelles of **SAIL-3** could be adsorbed at the oil–water interface of the emulsion. According to Rao and McClements, the particles (with peaks around 10 nm) do not adsorb at the interface boundary; on the other hand, peaks around 5000 nm correspond to larger lipid droplets that do not disrupt the creation process.^63^ Furthermore, the polydispersity index (PDI) is 0.18 ± 0.06. This result indicates a homogeneous system.^64,65^

## Conclusion

The use of synthesized surface-active amidequats with an alkoxymethyl substituent represents an innovative approach in the field of SAILs. This study demonstrates that these compounds exhibit unique properties that differentiate them from conventional surfactants. The incorporation of an additional long alkyl chain in the cation enhances the adsorption efficiency at the air–water interface and promotes micellization more effectively than surfactants with only one alkyl chain. Furthermore, synthesized amidequats can serve as alternatives to conventional anionic surfactants, and some of them, such as **SAIL-2**, -**3**, and -**5**, which exhibit lower CMC values, can replace the cationic surfactant didecyldimethylammonium chloride (DDAC) in emulsions. A further novelty in this work is the identification of **SAIL-3**, which contains 12 carbon atoms in its alkyl chain, as the most promising emulsifier. Analysis of o/w and w/o emulsions containing **SAIL-3** confirmed their stability, with the droplet size distribution ranging from 200 to 500 nm. Moreover, the o/w emulsion exhibited the characteristics of a so-called weak gel, indicating the potential of the compound to form colloidal systems. These results highlight the significance of surface-active amidequats with alkoxymethyl substituents, which, by forming stable emulsions, could potentially be used as delivery systems in the future.

## Supporting Information Available

^1^H NMR spectrum of dimethyl-*N*-[(2-octanamide)ethyl]octylokoxymethylammonium chloride (**SAIL-1**), ^13^C NMR spectrum of dimethyl-*N*-[(2-octanamide)ethyl]octylokoxymethylammonium chloride (**SAIL-1**), ^1^H NMR spectrum of decylokoxymethyldimethyl-*N*-[(2-octanamide)ethyl]ammonium chloride (**SAIL-2**), ^13^C NMR spectrum of decylokoxymethyldimethyl-*N*-[(2-octanamide)ethyl]ammonium chloride (**SAIL-2**), ^1^H NMR spectrum of cyclododecylokoxymethyldimethyl-*N*-[(2-octanamide)ethyl]ammonium chloride (**SAIL-5**), ^13^C NMR spectrum of cyclododecylokoxymethyldimethyl-*N*-[(2-octanamide)ethyl]ammonium chloride (**SAIL-5**), α and β dihedral angles of **SAIL-1** (for **SAIL2–4**, analogous C–C and C–O bonds were chosen), dependence of the dipole moment and dihedral angle (between aliphatic chains) as a function of aliphatic chain length ((CH_2_)_*n*_ for *n* = 8, 10, 12, or 14), dipole moments of **SAIL1–5** in vacuum and solvent (calculations were performed at the cam-B3LYP/6-311++G(d,p) level of theory; the IEFPCM model was used in the calculations for the solvent), dipole moments calculated for **SAIL1–5** using the B3LYP, cam-B3LYP, and ωB97x-D functionals and the 6-311++G(d,p) basis set, boiling points of chloromethylalkyl ethers at 1 hPa and their reaction yields, reaction yield and purity of the synthesized amidequats with alkoxymethyl substituents, dihedral angles between aliphatic chains for different energy states, dipole moments calculated for **SAIL1–5** at the cam-B3LYP/6-311++G(d,p) level of theory, dipole moments (in debyes) calculated for **SAIL1–5** (calculations were carried out for B3LYP, cam-B3LYP, and ωB97x-D functionals with the 6-311++G(d,p) or 6-31G(d,p) basis sets (marked with asterisks); calculations in the solvent were performed using the IEFPCM method), materials and methods of the preparation of SAILs, NMR analysis, and methodology of different scanning calorimetry (DSC), surface activity studies, foamability, atomic force microscopy (AFM), preparation of emulsions based on SAILs, characteristics of the emulsions, statistical analysis, computational methods (PDF)
