# Supplementary material for: Surface-Active Amidequats with an Alkoxymethyl Substituent: Synthesis, Analysis, and Preliminary Evaluation as Potential Emulsifiers and Substitutes for Conventional Surfactants
Source: Langmuir. 2025 Apr 16;41(16):10085–98. doi: 10.1021/acs.langmuir.4c04150 (PMC12044694; doi:10.1021/acs.langmuir.4c04150)
Supplement: Supplementary file 1 — la4c04150_si_001.pdf [file la4c04150_si_001.pdf]

ELECTRONIC SUPPLEMENTARY INFORMATION

Surface-active amidequats with alkoxymethyl substituent: synthesis, analysis and preliminary evaluation as potential emulsifiers and substitutes for conventional surfactants

Anna Syguda [†], *Marta Wojcieszak* [†,] *, *Sylwia Zięba* [‡], *Adam Mizera* [‡], *Andrzej Łapiński* [‡], *Jacek Różański* [†], *Alicja Putowska* [†], *Agnieszka Marcinkowska* [†], *Adam Grzywaczyk* [†], *Ewa Kaczorek* [†] *and Katarzyna Materna* [†,] *

[†] Institute of Chemical Technology and Engineering, Poznan University of Technology, Berdychowo 4, Poznan 60–965, Poland

[‡] Institute of Molecular Physics, Polish Academy of Sciences, M. Smoluchowskiego 17, Poznan, 60-179, Poland

*corresponding author: katarzyna.materna@put.poznan.pl marta.wojcieszak@put.poznan.pl

Number of pages: 24

Number of figures: 12

Number of tables: 5

A table of contents:

Figure S.1. ^{1}H NMR spectrum of dimethyl-*N*-[(2-octanamide)ethyl]octylokoxymethylammonium chloride (**SAIL-1**)………………………………………………………………………………...S3
Figure S.2. ^{13}C NMR spectrum of dimethyl-*N*-[(2-octanamide)ethyl]octylokoxymethylammonium chloride (**SAIL-1**)………………………………………………………………………………...S4
Figure S.3. ^{1}H NMR spectrum of decylokoxymethyldimethyl-*N*-[(2-octanamide)ethyl]ammonium chloride (**SAIL-2**)………………………………………………………………………………...S5

Figure S.4. ^{13}C NMR spectrum of decylokoxymethyldimethyl-*N*-[(2-octanamide)ethyl]ammonium chloride (**SAIL-2**)...S6
Figure S.5. ^{1}H NMR spectrum of cyclododecylokoxymethyldimethyl-*N*-[(2-octanamide)ethyl]ammonium chloride (**SAIL-5**)...S7
Figure S.6. ^{13}C NMR spectrum of cyclododecylokoxymethyldimethyl-*N*-[(2-octanamide)ethyl]ammonium chloride (**SAIL-5**)...S8
Figure S.7. DSC thermograms of synthesised SAILs...S10
Figure S.8. The α and β dihedral angles in **SAIL-1**. Note: for **SAILs 2-4**, analogous C-C and C-O bonds were chosen...S10
Figure S.9. Dependence of dipole moment and dihedral angle (between aliphatic chains) as a function of aliphatic chain length; $(CH_2)_n$ for n=8, 10, 12, 14...S11
Figure S.10. Dipole moments of **SAILs 1-5** in vacuum and solvent. Note: Calculations were performed at the cam-B3LYP/6-311++G(d,p) level of theory. The IEFPCM model was used in the calculations for the solvent...S12
Figure S.11. Dipole moments calculated for **SAILs 1-5** using the functional B3LYP, cam-B3LYP, and ωB97x-D and the basis set 6-311++G(d,p)...S13
Table S1. Boiling points of chloromethylalkyl ethers at 1 hPa and their reaction yields...S14
Table S2. Reaction yield and purity of the synthesized amidequats with alkoxymethyl substituent...S14
Table S3. Dihedral angle (°) between aliphatic chains for different energy states...S14
Table S4. Dipole moments calculated for **SAILs 1-5** at the cam-B3LYP/6-311++G(d,p) level of theory...S14
Table S5. Dipole moments (in Debye) calculated for **SAILs 1-5**. Calculations were carried out for B3LYP, cam-B3LYP, and ωB97x-D functional with the base set 6-311++G(d,p) or 6-31G(d,p) (marked with *). Calculations in the solvent were performed using the IEFPCM method...S14
Materials...S15
Methods...S15
Preparation...S15
Synthesis of *N*-[(2-dimethylamino)ethyl]octanamide...S15
Synthesis of chloromethylalkyl ethers...S16
Synthesis of alkoxymethyldimethyl-*N*-[(2-octanamide)ethyl]ammonium chlorides...S16
Synthesis of sodium caprylate...S17
NMR analysis...S17
Different Scanning Calorimetry (DSC)...S17
Surface activity studies...S18
Figure S.12. Image of the CA of a drop on a hydrophobic (paraffin) surface...S18
Foamability...S21
Atomic force microscopy (AFM)...S21
Preparation of emulsions based on SAILs...S21
Method of preparation oil-in-water emulsion...S21
Method of preparation water-in-oil emulsion...S22
Characteristics of the emulsions...S22
Statistical analysis...S23
Computational methods...S23

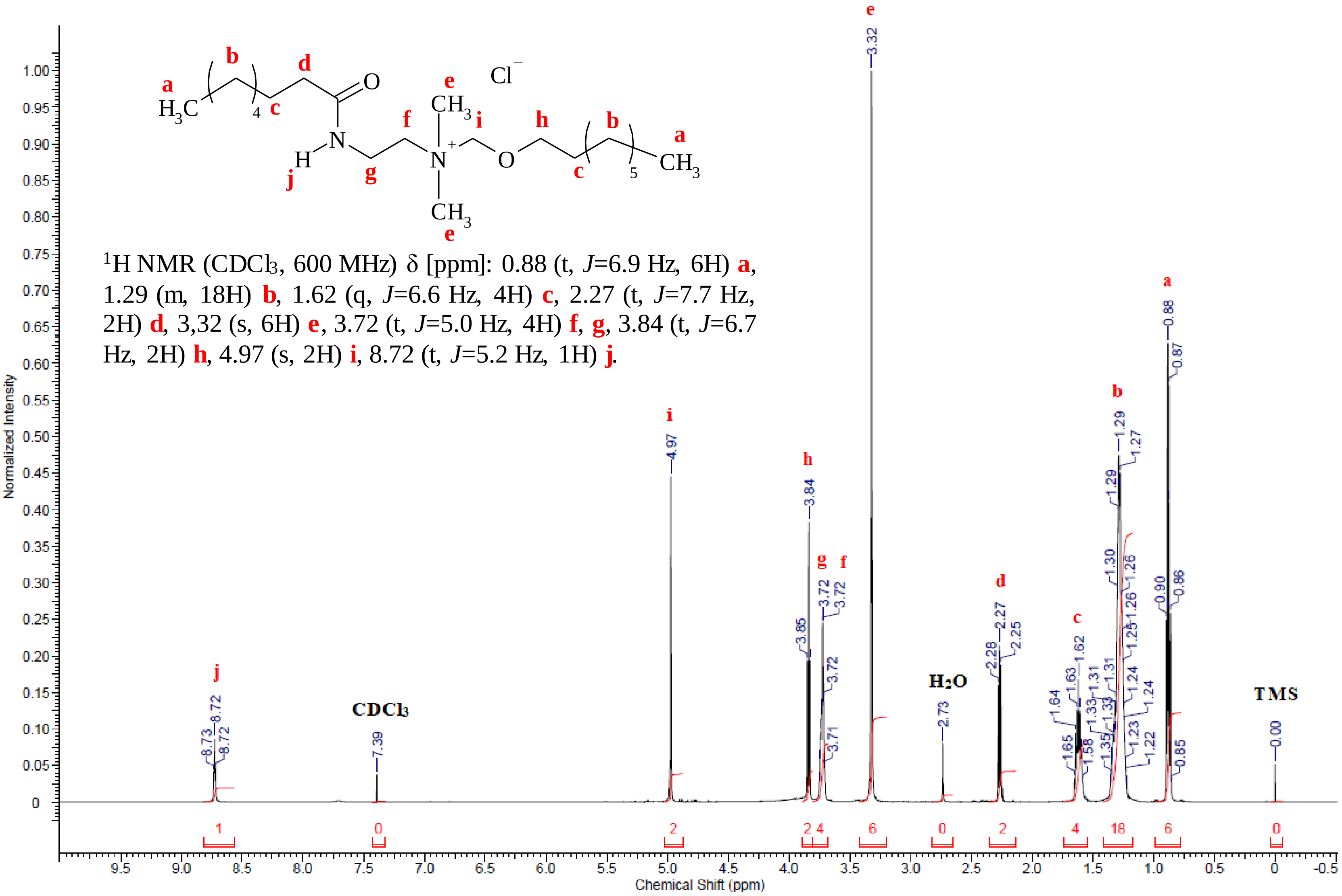

Figure S.1. ¹H NMR spectrum of dimethyl-*N*-[(2-octanamide)ethyl]octylokoxymethylammonium chloride (**SAIL-1**)

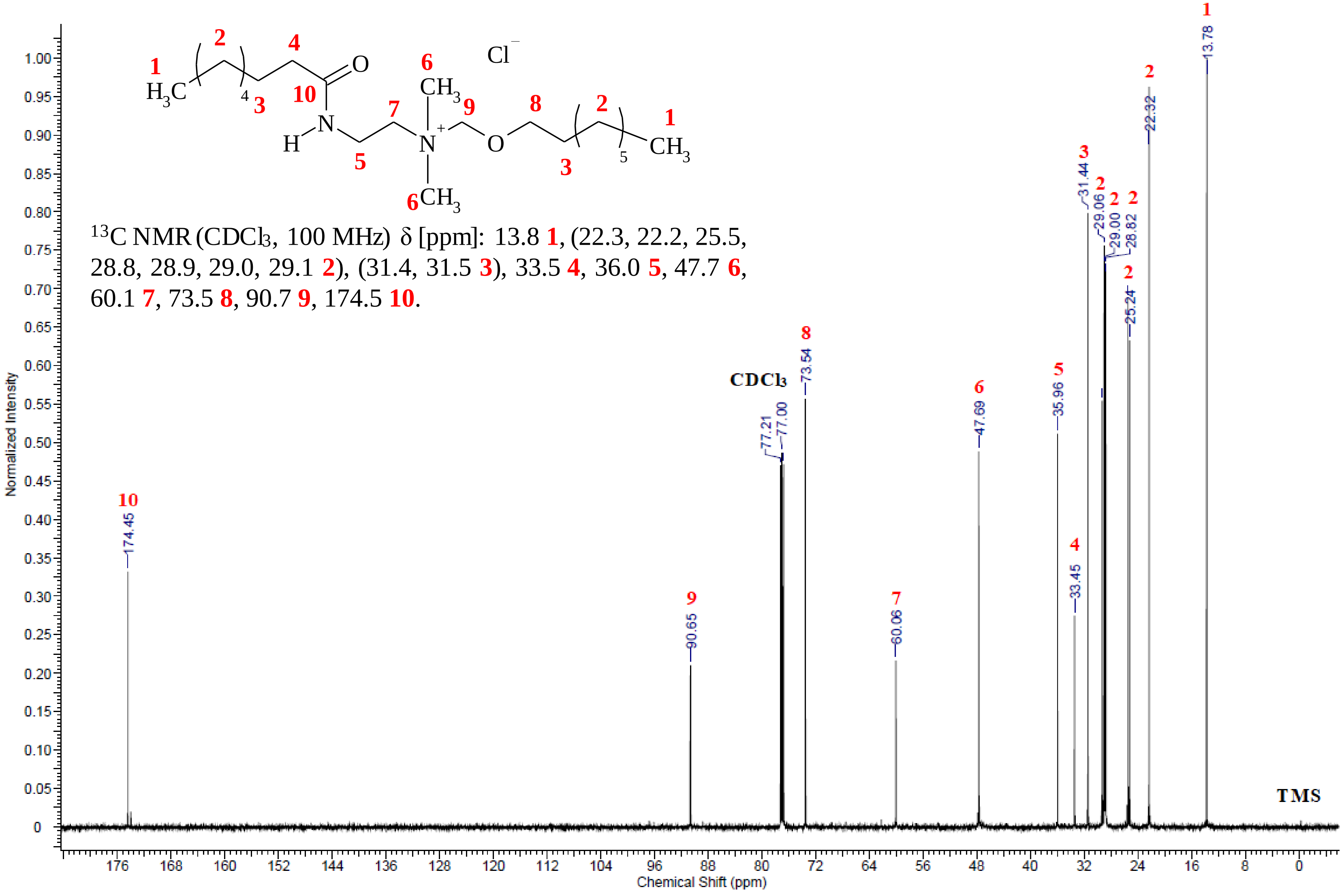

Figure S.2. ^{13}C NMR spectrum of dimethyl-*N*-[(2-octanamide)ethyl]octylokoxymethylammonium chloride (**SAIL-1**)

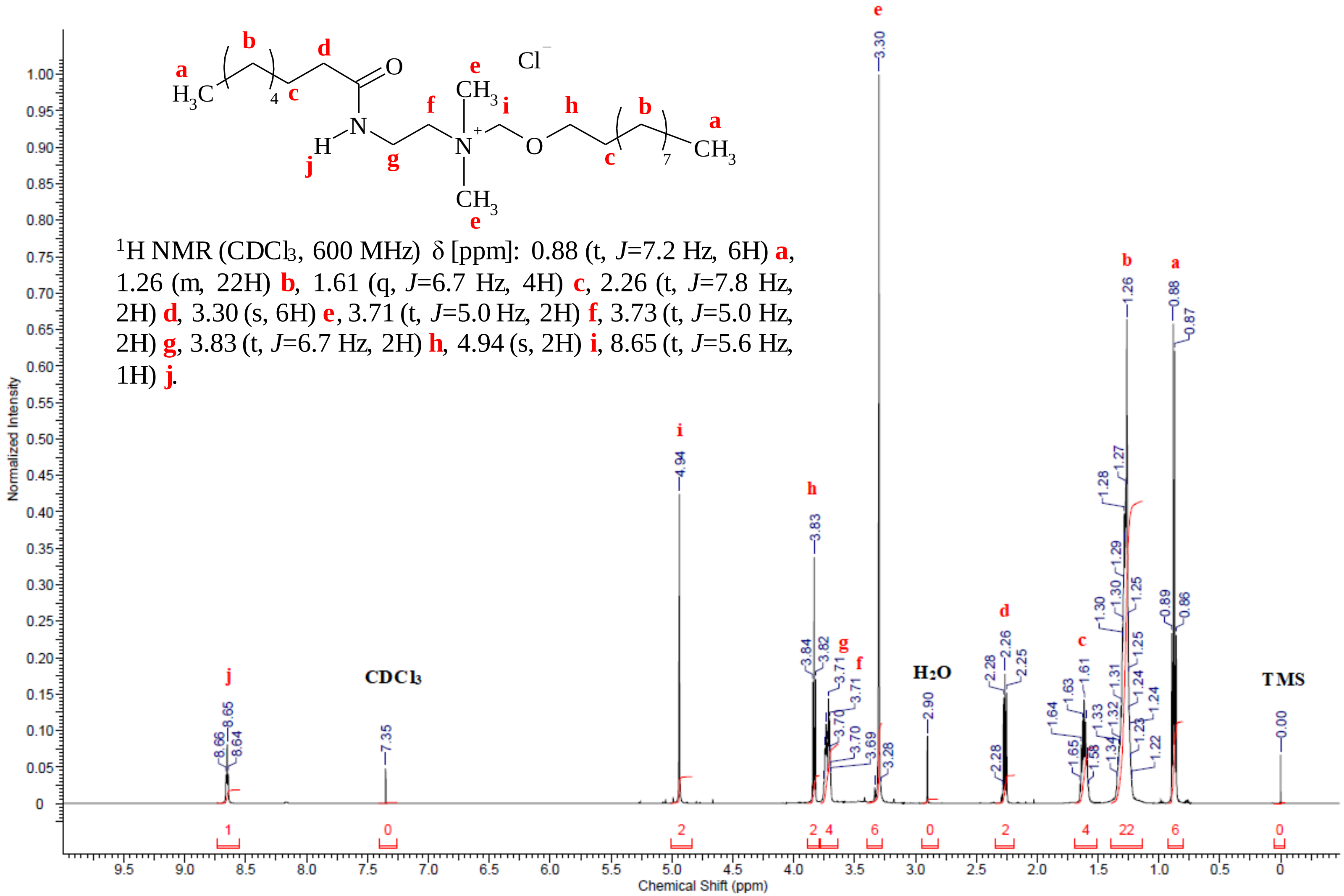

Figure S.3. ^{1}H NMR spectrum of decylokoxymethyldimethyl-*N*-[(2-octanamide)ethyl]ammonium chloride (**SAIL-2**)

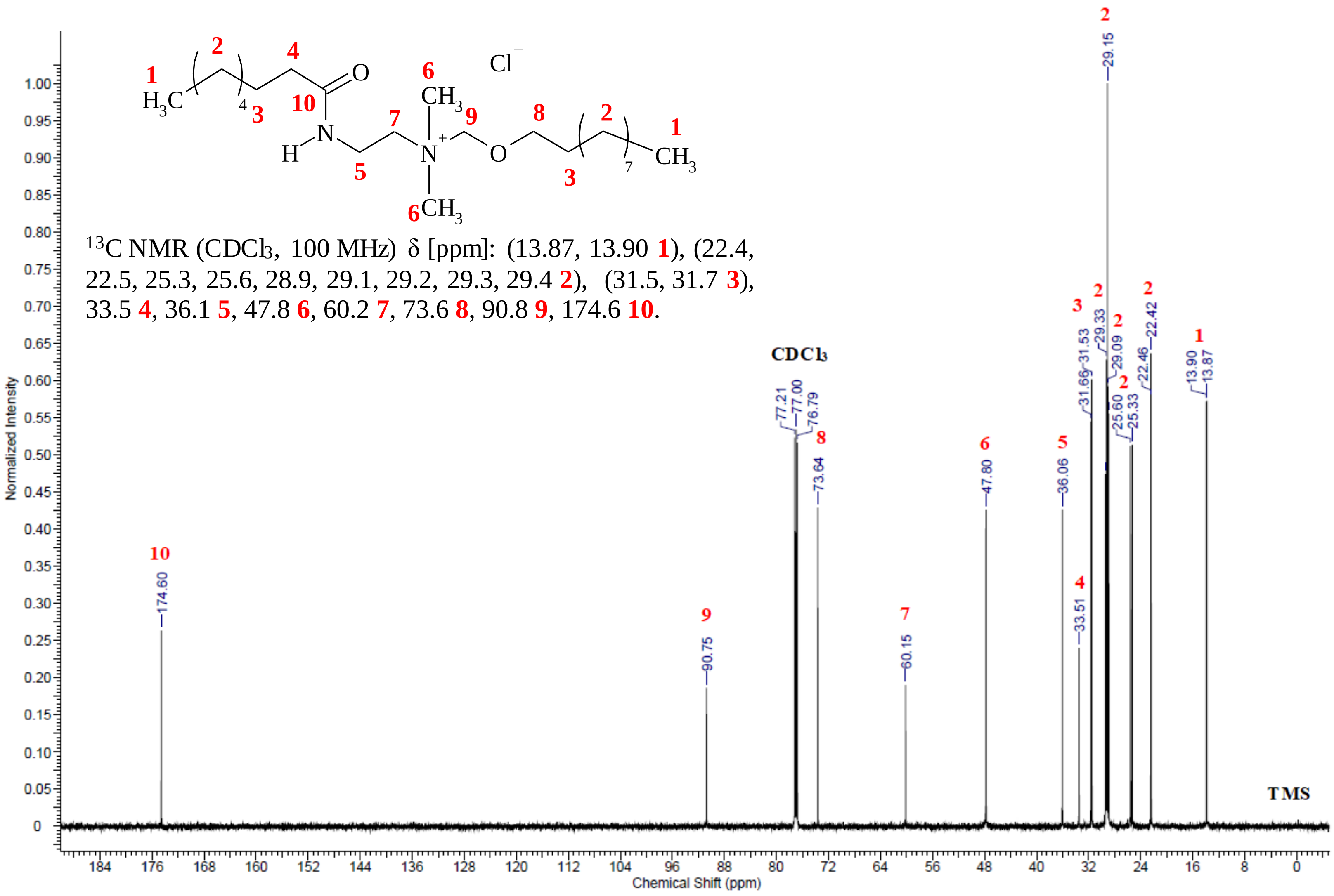

Figure S.4. ^{13}C NMR spectrum of decylokoxymethyldimethyl-*N*-[(2-octanamide)ethyl]ammonium chloride (**SAIL-2**)

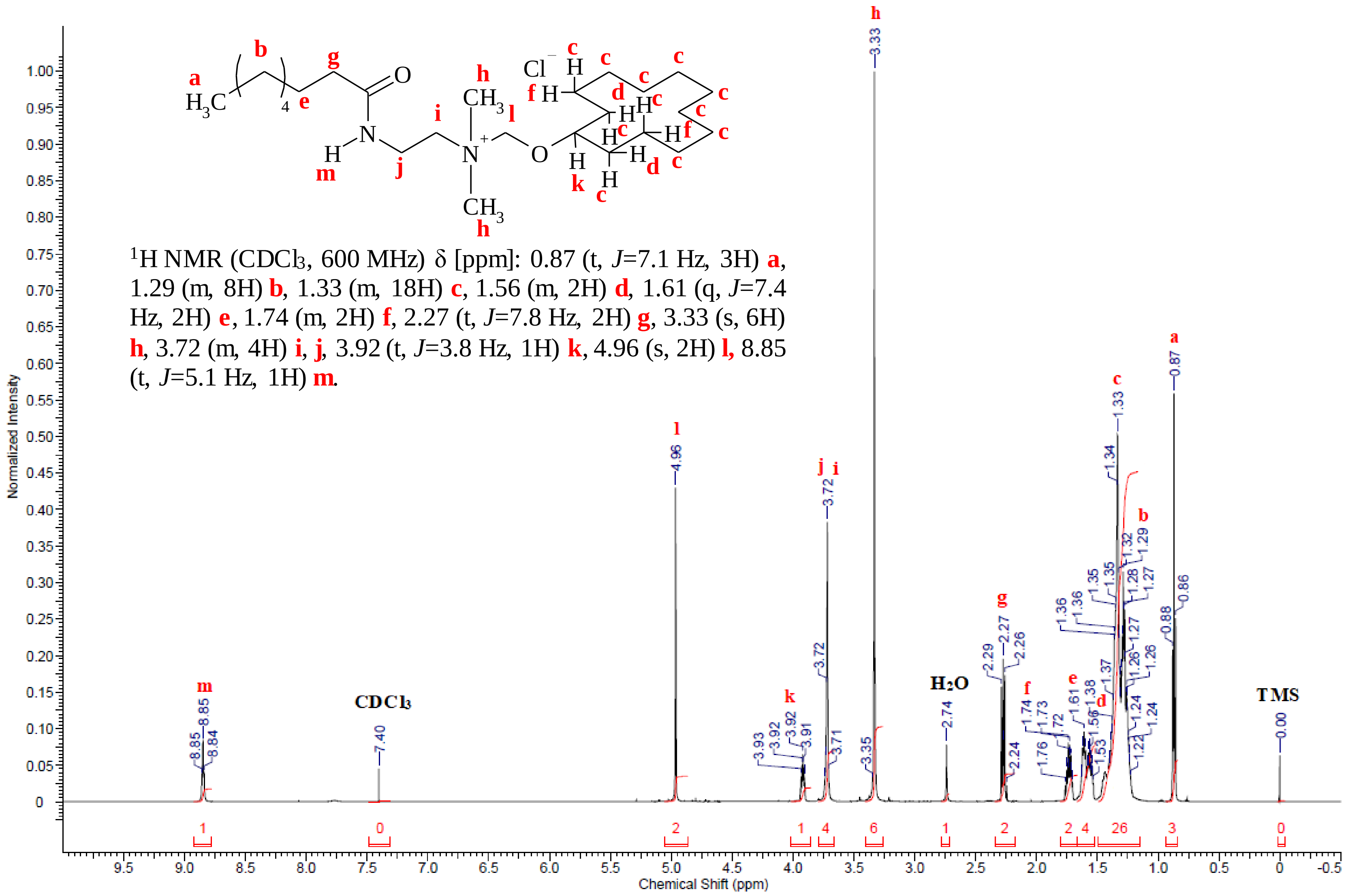

Figure S.5. ^{1}H NMR spectrum of cyclododecylokoxymethyldimethyl-*N*-[(2-octanamide)ethyl]ammonium chloride (**SAIL-5**)

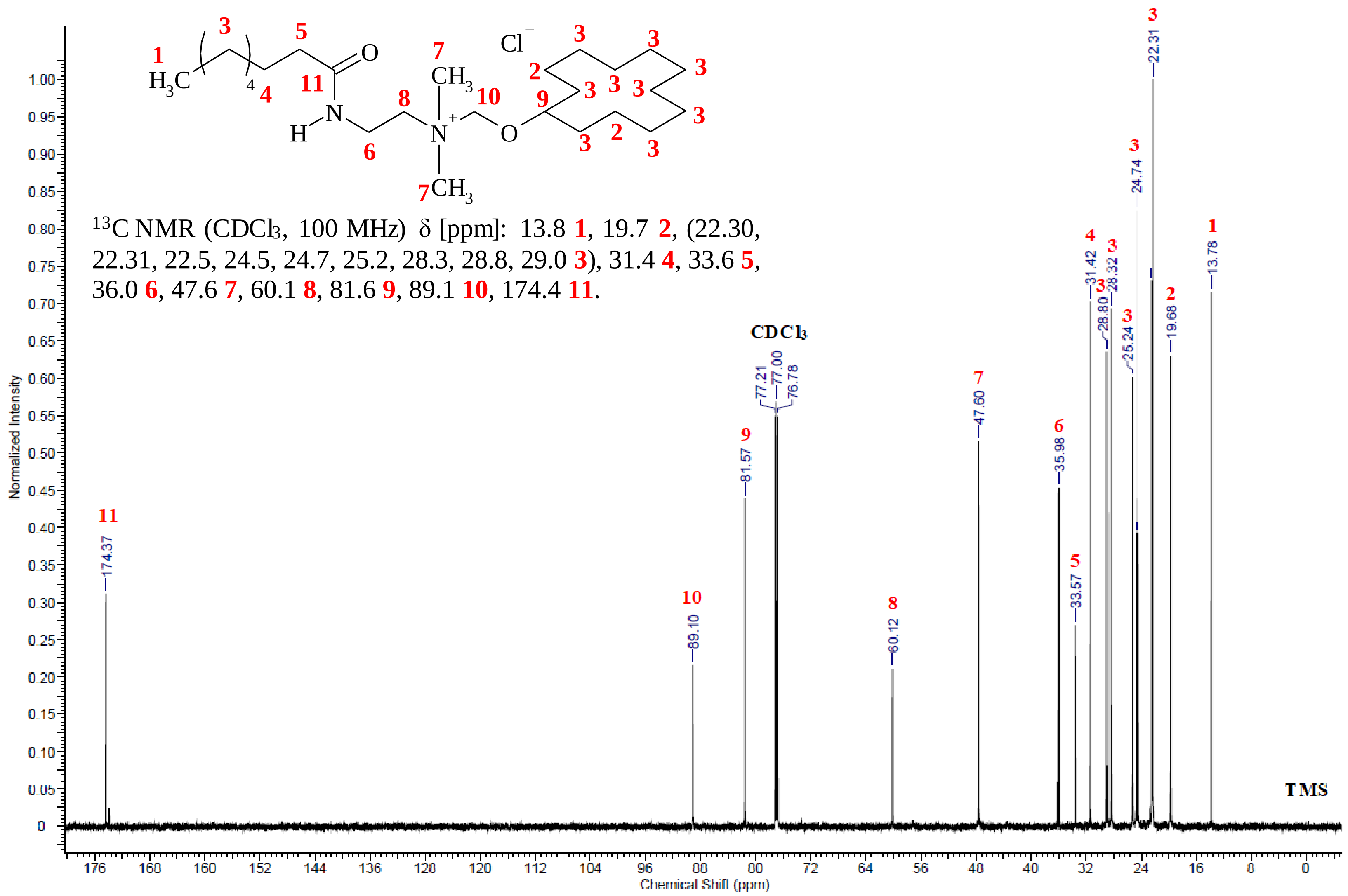

Figure S.6. ^{13}C NMR spectrum of cyclododecylokoxymethyldimethyl-*N*-[(2-octanamide)ethyl]ammonium chloride (**SAIL-5**)

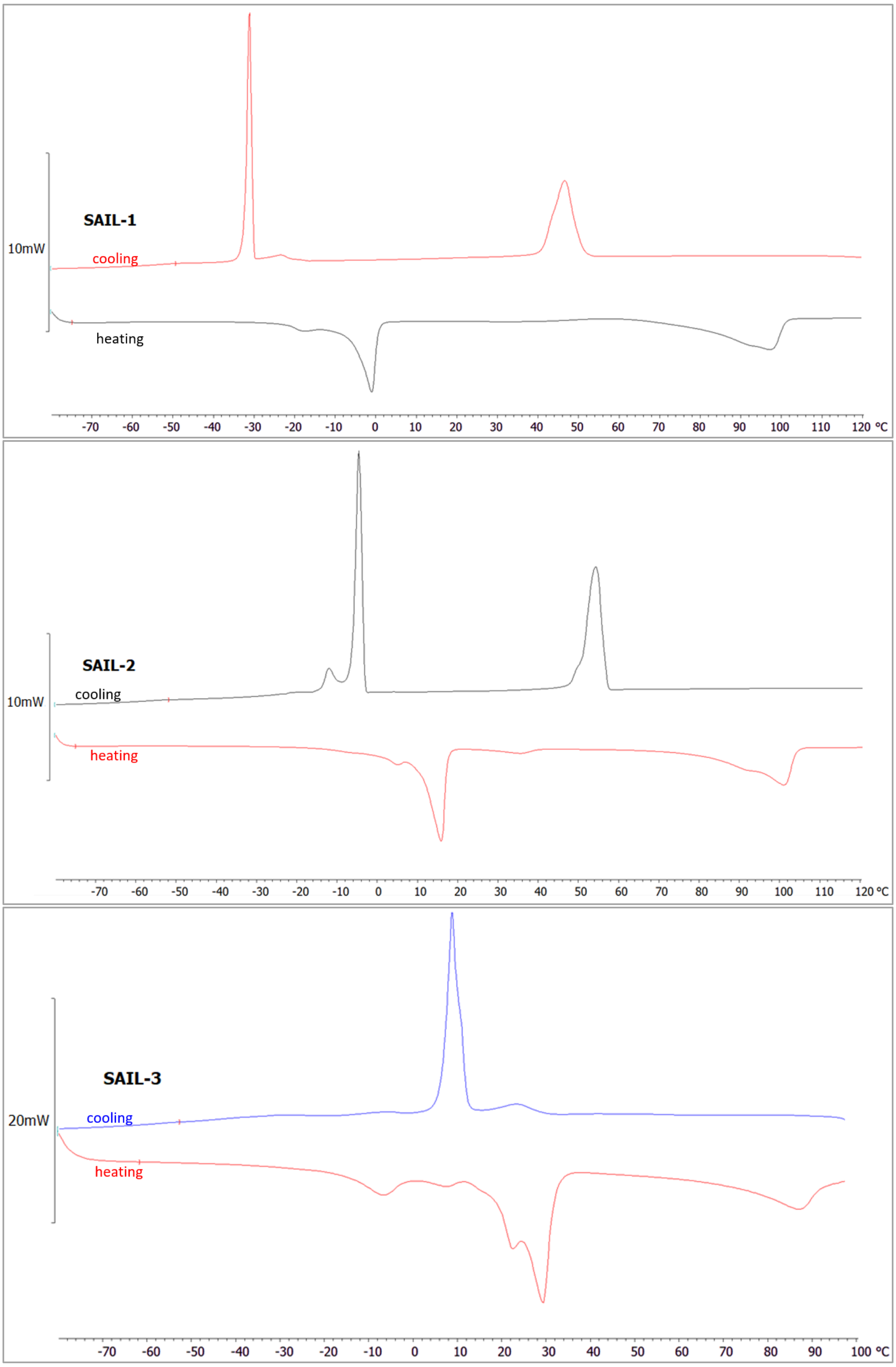
SAIL-1
10mW
cooling
heating
-70 -60 -50 -40 -30 -20 -10 0 10 20 30 40 50 60 70 80 90 100 110 120 °C
SAIL-2
10mW
cooling
heating
-70 -60 -50 -40 -30 -20 -10 0 10 20 30 40 50 60 70 80 90 100 110 120 °C
SAIL-3
20mW
cooling
heating
-70 -60 -50 -40 -30 -20 -10 0 10 20 30 40 50 60 70 80 90 100 °C

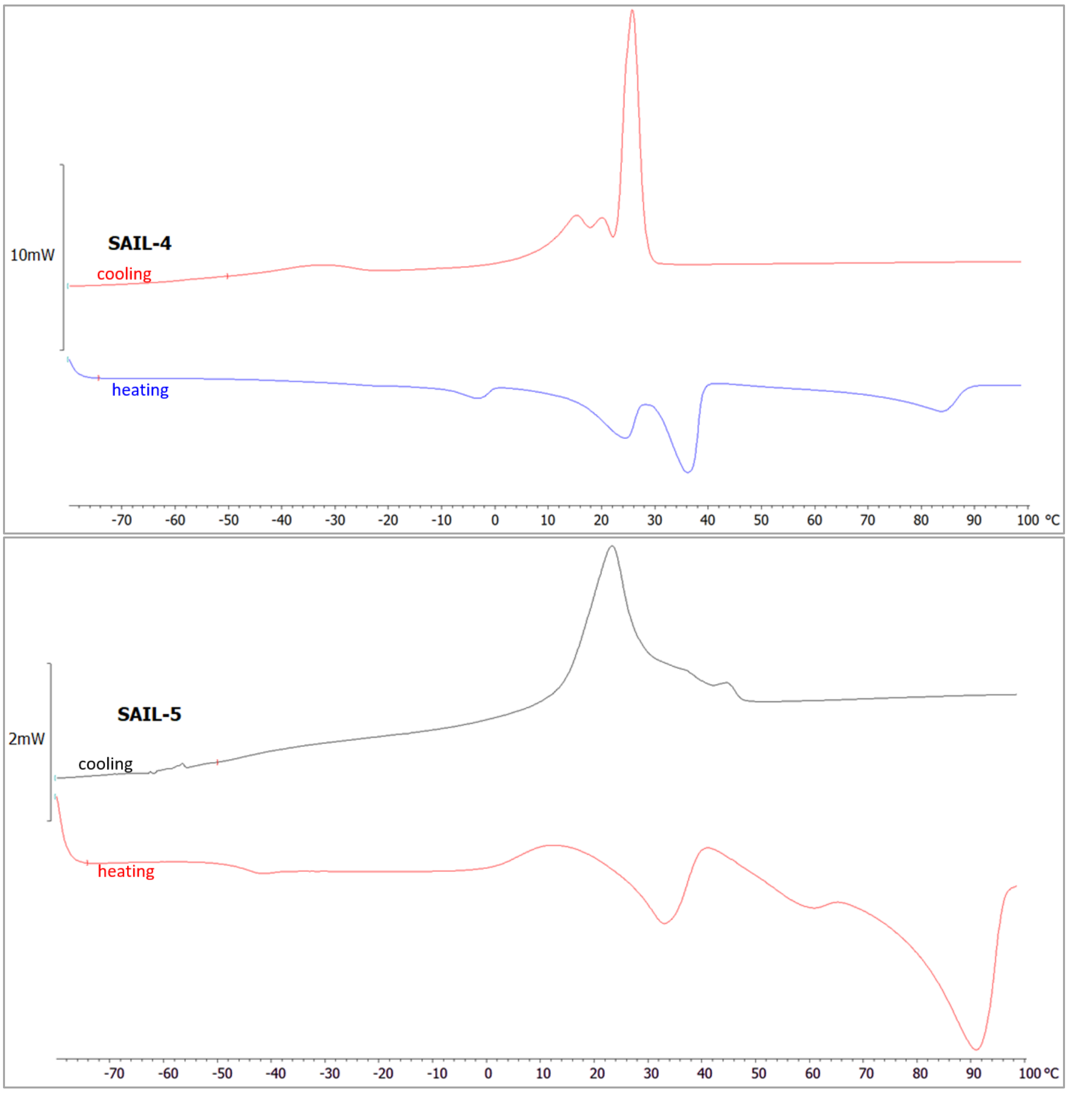

Figure S.7. DSC thermograms of synthesised SAILs

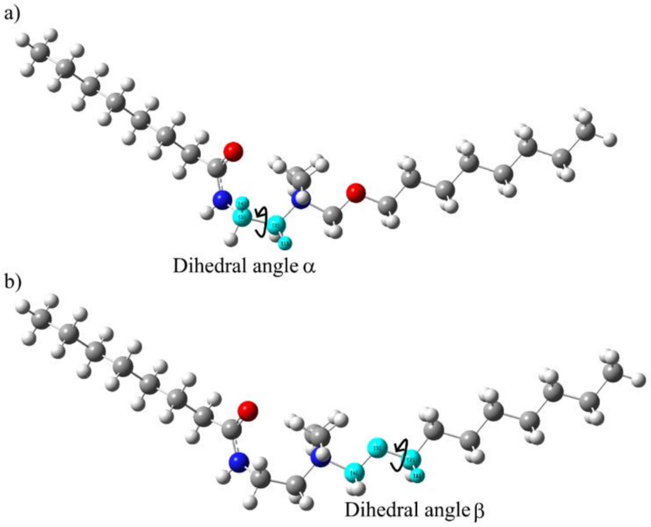

Figure S.8. The α and β dihedral angles in **SAIL-1**. Note: for **SAILs 2-4**, analogous C-C and C-O bonds were chosen

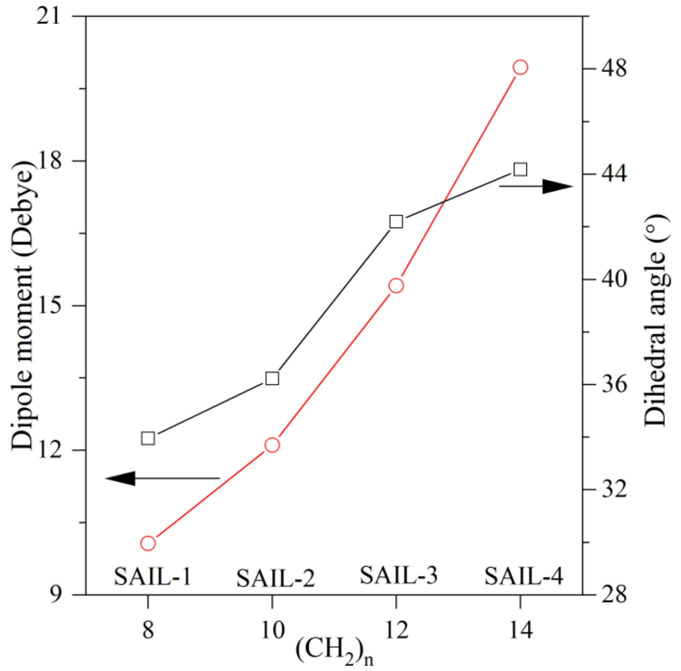

Figure S.9. Dependence of dipole moment and dihedral angle (between aliphatic chains) as a function of aliphatic chain length; $(CH_2)_n$ for n=8, 10, 12, 14

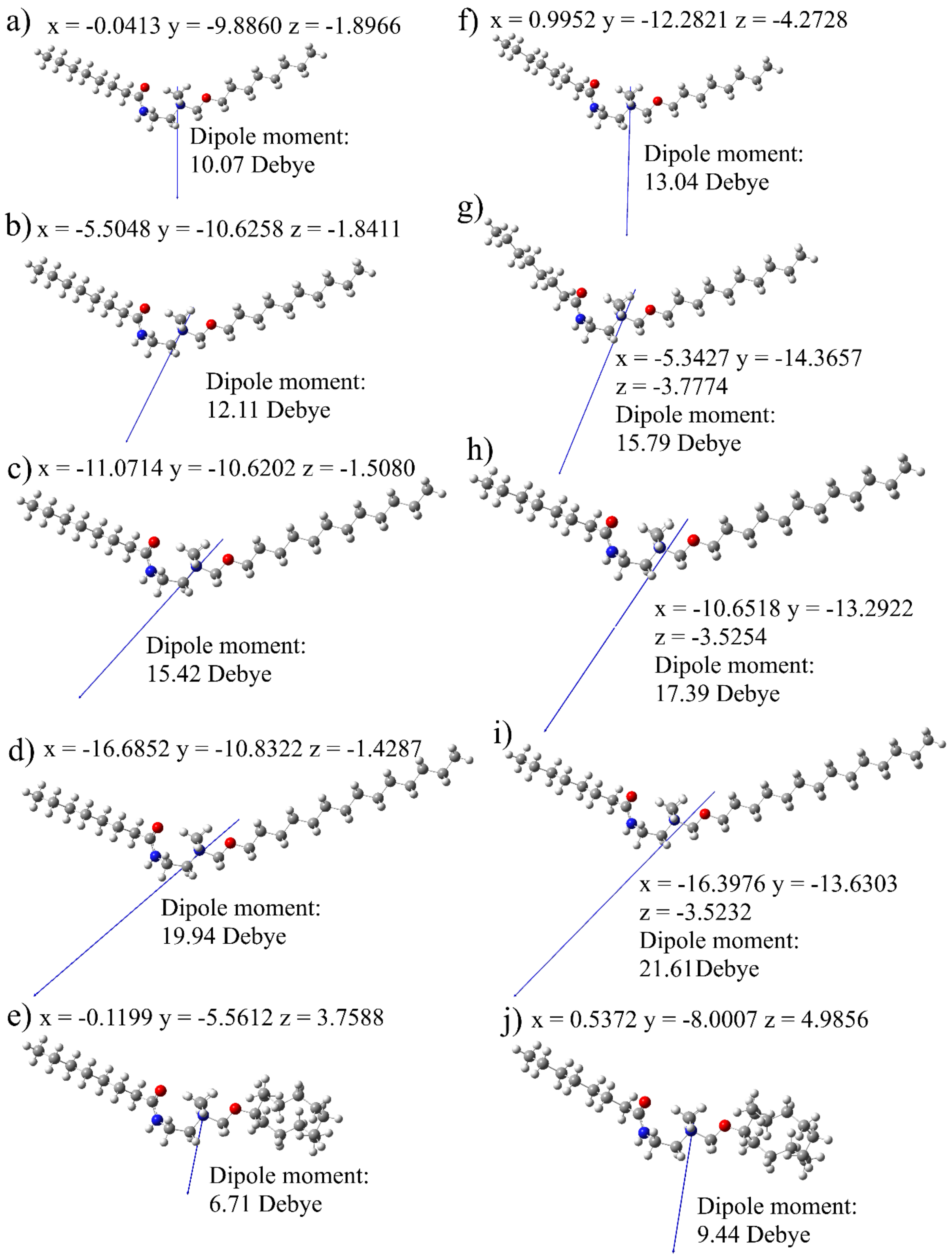

Figure S.10. Dipole moments of **SAILs 1-5** in vacuum and solvent. Note: Calculations were performed at the cam-B3LYP/6-311++G(d,p) level of theory. The IEFPCM model was used in the calculations for the solvent

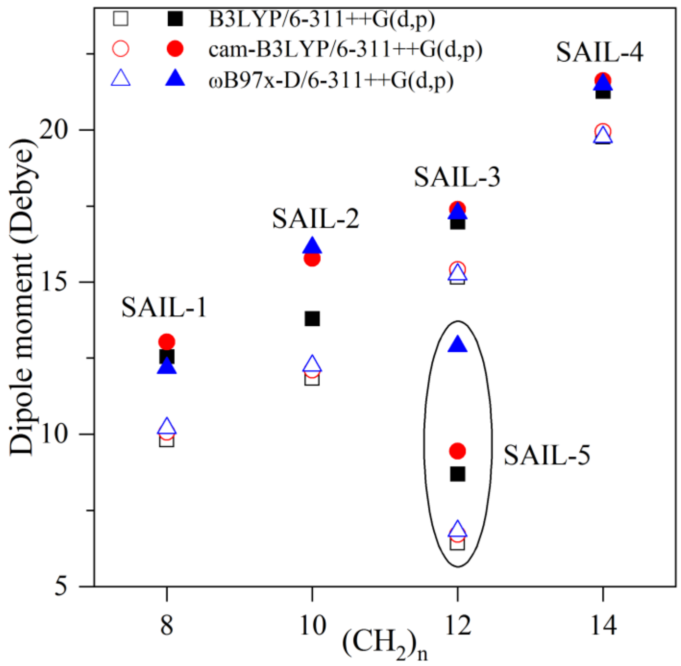

Figure S.11. Dipole moments calculated for **SAILs 1-5** using the functional B3LYP, cam-B3LYP, and ωB97x-D and the basis set 6-311++G(d,p)

Table S1. Boiling points of chloromethylalkyl ethers at 1 hPa and their reaction yields

Chloomethylalkyl ether	Boiling point [°C] at 1 hPa	Yield [%]
$C_8H_{17}OCH_2Cl$	82-83	90
$C_{10}H_{21}CH_2Cl$	105-106	93
$C_{12}H_{25}CH_2Cl$	126-127	95
$C_{14}H_{29}CH_2Cl$	144-145	98
$C_{12}H_{23}$(cyclic)CH_2Cl	138-139	97

Table S2. Reaction yield and purity of the synthesized amidequats with alkoxymethyl substituent

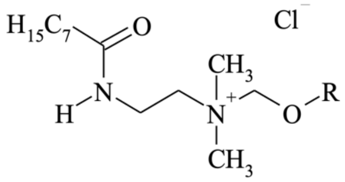

Abbreviation	R	Purity [%]	Yield [%]
**SAIL-1**	C_8H_{17}	95.0	85
**SAIL-2**	$C_{10}H_{21}$	97.0	89
**SAIL-3**	$C_{12}H_{25}$	98.0	92
**SAIL-4**	$C_{14}H_{29}$	95.0	99
**SAIL-5**	$C_{12}H_{23}$(cyclic)	99.0	92

Table S3. Dihedral angle (°) between aliphatic chains for different energy states

Abbreviation	GS1	TS1β	GS2β	TS2β	GS3β	TS3β
**SAIL-1**	33.94	54.44	97.69	179.39	110.87	91.30
**SAIL-2**	36.18	59.93	95.25	176.85	113.28	93.67
**SAIL-3**	42.19	46.49	80.21	165.72	123.32	103.10
**SAIL-4**	44.19	44.56	71.94	163.92	125.09	104.89

Table S4. Dipole moments calculated for **SAILs 1-5** at the cam-B3LYP/6-311++G(d,p) level of theory

Abbreviation	GS1	GS2α	GS2β	GS3β
**SAIL-1**	10.07	6.99	6.73	7.90
**SAIL-2**	12.11	6.59	8.17	9.05
**SAIL-3**	15.42	10.30	11.63	11.64
**SAIL-4**	19.94	14.71	16.50	16.45

Table S5. Dipole moments (in Debye) calculated for **SAILs 1-5**. Calculations were carried out for B3LYP, cam-B3LYP, and ωB97x-D functional with the base set 6-311++G(d,p) or 6-31G(d,p) (marked with *). Calculations in the solvent were performed using the IEFPCM method

Abbreviation	in vacuum			in solution		
	B3LYP	cam-B3LYP	ωB97x-D	B3LYP	cam-B3LYP	ωB97x-D
**SAIL-1**	9.81	10.07	10.21	12.56	13.04	12.18
**SAIL-2**	11.84	12.11	12.25*	13.81	15.79*	16.14*
**SAIL-3**	15.16	15.42	15.25	16.98	17.39	17.27
**SAIL-4**	19.77	19.94	19.76	21.27	21.61	21.49*
**SAIL-5**	6.43	6.71	6.83	8.70	9.44	12.90

Materials

(2-Dimethylamino)ethylamine (CAS 108-00-9) 98%, caprylic acid (CAS 124-07-2) 98%, *p*-toluenosulfonic acid (CAS 6192-52-5) 98,5%, octan-1-ol (CAS 111-87-5) 99%, decan-1-ol (CAS 112-30-1) 98%, dodecan-1-ol (CAS 112-53-8) 98%, tetradecan-1-ol (CAS 112-72-1) 97%, cyclododecanol (CAS 1724-39-6) 99%, 1,3,5-trioksane (CAS 110-88-3) 99%, cetyl alcohol (CAS 36653-82-4) 99.5%, citric acid (CAS 77-92-9) 99.5%, liquid paraffin (CAS 8012-95-1) 100%, white petrolatum (CAS 8009-03-8) 99%, reagents for two-phase system titration: [dimidium bromide (CAS 95-518-67-2) 95%, patent blue V sodium salt (CAS 20262-76-4) 97%, sodium dodecylsulfate(VI) (CAS 151-21-3) 98%] were purchased from Merck. Hydrochloric acid (CAS 7647-01-0) 36%, sulfuric(VI) acid (CAS 7664-93-9) 96%, sodium bicarbonate (CAS 144-55-8) 99%, anhydrous sodium sulfate(VI) (CAS 7757-82-6) 99+% and solvents: toluene (CAS 108-88-3) 99%, acetone (CAS 67-64-1) 99%, hexane (CAS 110-54-3) 99%, ethyl acetate (CAS 141-78-6) 99%, chloroform (CAS 67-66-3) 98.5% were purchased from Avantor. Isopropyl palmitate (CAS 142-91-6) 97% was purchased from TCI. Cetearyl alcohol (CAS 67762-27-0) (which is mixture of cetyl and stearyl alcohol) was purchased from PCC Exol SA. Glycerol monostearate (CAS 31566-31-1) > 95% was purchased from ThermoFisher. Sunflower oil was purchased from Ecol.

Methods

<u>Preparation</u>

Synthesis of *N*-[(2-dimethylamino)ethyl]octanamide

In a 250 mL flask, 20 g of (2-dimethylaminoethyl)amine was dissolved in 20 mL of toluene. Then, a stoichiometric amount of caprylic acid, along with *p*-toluenesulfonic acid as a catalyst (10 mol %), was added to the mixture, also in 20 mL of toluene. The reaction mixture was refluxed at boiling temperature until the complete removal of the water formed during the reaction, using a Dean–Stark apparatus. Next, the organic phase was washed with 20 mL of 10% sodium bicarbonate solution. The phases were separated, and the organic layer was dried for 20 minutes over anhydrous sodium sulfate(VI). The precipitate was filtered off, and the

solvent was evaporated using a rotary evaporator under vacuum. Finally, the product was purified by distillation under reduced pressure.

Synthesis of chloromethylalkyl ethers

In a 250 mL round-bottomed three-necked flask equipped with a magnetic stirrer, thermometer, and ceramic frit, 50 g of the appropriate alcohol and 1,3,5-trioksane (with a 5% excess over the stoichiometric amount) were placed. For alcohols that were solid (dodecan-1-ol, tetradecan-1-ol, cyclododecanol), 100 mL of toluene was used as a solvent. The resulting suspension was saturated with dried hydrogen chloride. Gaseous HCl was generated by the continuous dropwise addition of concentrated sulfuric(VI) acid to a solution of concentrated hydrochloric acid, and then dried by passing it through a system of scrubbers with concentrated sulfuric(VI) acid as the drying agent. Unreacted hydrogen chloride was absorbed in a scrubber packed with water. The temperature in the reaction flask was maintained in the range of 15-20 °C. After the reaction was completed, as evidenced by the lack of hydrogen chloride absorption and the clarification of the reaction mixture, the organic layer was separated and dried over anhydrous sodium sulfate(VI) for 24 hours. The absorbed hydrogen chloride was then stripped from the organic layer by aeration with dried air. In reactions carried out with a solvent, the solvent was removed by evaporation under reduced pressure. In the final stage, the crude chloromethylalkyl ethers were purified by vacuum distillation.

Synthesis of alkoxymethyldimethyl-*N*-[(2-octanamide)ethyl]ammonium chlorides

Five 100 mL round-bottom flasks were each filled with 5 g of *N*-[(2-dimethylamino)ethyl]octanamide, 10 mL of cold acetone, and a stoichiometric amount of appropriate chloromethylalkyl ether. The resulting mixtures were vigorously stirred for 20 min at room temperature. After 20 min, the acetone was completely evaporated from the post-reaction mixtures using a rotary evaporator, and the raw products were placed in the refrigerator for 30 min. Then, 10 mL of cold ethyl acetate was added to the flasks with the products. Next,

the products were vacuum-filtered and washed with cold ethyl acetate. Finally, the alkoxymethyldimethyl-*N*-[(2-octanamide)ethyl]ammonium chlorides were dried in a vacuum oven.

Synthesis of sodium caprylate

In a 250 mL flask, 5 g of caprylic acid was placed, and then a stoichiometric amount of sodium bicarbonate and 50 mL of deionized water were added. The mixture was heated on a water bath at 100 °C until the sodium bicarbonate had completely reacted and carbon dioxide had ceased to be released. Then the reaction mixture was then concentrated on a rotary evaporator, evaporating about 90% of the water. In the next step, the flask with the product was cooled in a refrigerator, and the product was subsequently filtered cold, washed with cold water, then with acetone, and dried in a vacuum desiccator at 60 °C. A white precipitate was obtained with a yield of 95%.

NMR analysis

Nuclear magnetic resonance (NMR) spectra (proton – ^{1}H and carbon – ^{13}C) were recorded at 25 °C in a Bruker Avance 600 MHz spectrometer operating at 600 MHz for ^{1}H nuclei and 100 MHz for ^{13}C nuclei. Samples were dissolved in $CDCl_3$ using tetramethylsilane (TMS) as an internal reference (δ = 0.00 ppm).

Different Scanning Calorimetry (DSC)

The melting (T_m) and crystallization (T_c) temperatures of synthesized materials were determined by differential scanning calorimetry using a DSC1 instrument (Mettler-Toledo, Greifensee, Switzerland). Samples of 5 - 10 mg were weighed in aluminum crucibles and placed in the apparatus chamber through which inert gas (argon) was passed at a 50 ml/min rate. The measurements were carried out in the temperature range of -70 – 120 °C (and for sodium octanoate -70 – 300 °C) with a heating/cooling rate of 10 °C/min. The sample was heated to 120

°C in the first cycle to remove water from the synthesis. The melting temperatures (T_m) and cold crystallization temperatures (T_{cc}) were determined from the second heating cycle, whereas crystallization temperatures (T_c) were determined from the second cooling cycle on the DSC thermograms.

Surface activity studies

Surface activity was investigated utilizing a DSA 100 analyzer from Krüss, Germany, with an accuracy of ± 0.01 mN/m. The determination of surface tension (γ) employed the *Pendant Drop Method*, analyzing the drop profile in accordance with Laplace's equation. From the obtained measurement results, various parameters were derived, including critical micelle concentration (CMC), surface tension at the CMC (γ_{CMC}), Gibbs free energy of adsorption (ΔG^0_{ads}), surface pressure at the CMC (Π_{CMC}), surface excess concentrations at the saturated interface (Γ_{max}), minimum surface occupied by a molecule at the interface (A_{min}), and adsorption efficiency (pC_{20}). Wettability, on the other hand, was assessed through contact angle (CA) measurements using the *Sessile Drop Method*. The basis for determining the CA values was the image of the drop placed on the tested hydrophobic paraffin surface (see Figure S.12.). Nevertheless, when meticulously describing the measurement process itself, several steps should be selected. A drop of the solution of the compound in question was placed on the prepared surface using a syringe in such a precise and accurate way that the drop almost 'settles' on the paraffin. Then, based on the sessile drop method, the shape of the drop was adjusted. After the actual shape of the deposited drop and the contact line were determined, the contour was adapted to the mathematical model used to calculate the CA.

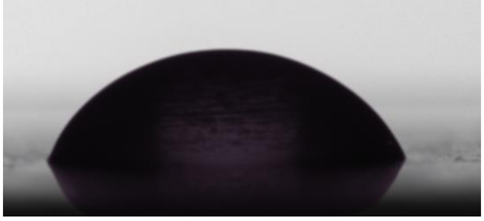

Figure S.12. Image of the CA of a drop on a hydrophobic (paraffin) surface

The precise equations for computing the parameters ΔG^0_{ads}, A_{min}, π_{CMC} pC_{20}, and Γ_{max} are elucidated in previous publications [11,12,30]. However, in order to make our work more readily perceivable, we include below the equations relating to the mentioned parameters.

Gibbs energy (ΔG^0_{ads}) which characterizes the interactions between the bulk phase and the surface phase:

$$\Delta G^0{}_{ads} = -nRTlna$$

where:

n is the number of solute species at the interface and it therefore set as n = 2,

R is gas constant,

T is absolute temperature.

The a value is a parameter of the Szyszkowski equation:

$$\gamma = \gamma_0[1\text{-bln}\left(\frac{c}{a}+1\right)]$$

where:

γ_0 is the surface tension of the solvent.

Surface excess concentrations (Γ_{max}) were calculated from the slope of the linear portion of the γ-log C plots (Fig. 4) using the Gibbs isotherm:

$$\Gamma_{max} = -\frac{1}{nRT}\left(\frac{d\gamma}{d\ln C}\right)$$

where:

R is gas constant,

T is absolute temperature,

n is the number of solute species at the interface and it therefore set as n = 2,

C is concentration of salts.

From Γ_{max} the minimum surface occupied by a molecule at the interface A_{min} can be calculated from equation:

$$A_{min} = \frac{1}{\Gamma_{max} N_A}$$

where:

N_A is the Avogadro number.

The precise equations for computing the parameters ΔG^0_{ads}, A_{min}, and Γ_{max} are elucidated in previous publications

Surface pressure at the CMC (π_{CMC}) can be calculated from equation:

$$\pi_{CMC} = \gamma_0 - \gamma_{CMC}$$

where:

γ_0 is surface tension of pure water which equals 72.8 mN m^{-1} (at under measurement conditions, 25 °C).

Adsorption efficiency (pC_{20}):

$$pC_{20} = -\log C_{20}$$

Foamability

Foaming ability (FA) and foam durability index (FDI), for series of synthetized SAILs were studied with a concentration according to the CMC at 25 °C. All the individual steps of conducting the study and the formulas used to calculate the FA and FDI values were followed according to the methodology presented in our recent work.

Atomic force microscopy (AFM)

In a first step, aqueous solutions of the synthesized SAILs were prepared. The solutions used for the analyses were those with concentrations before CMC. Then a small volume (25 µl) of the solutions of SAILs was applied to a 1x1 cm piece of freshly cleaned mica using a syringe. These prepared samples were allowed to air dry for 24 hours, with precautions taken to prevent potential contamination from the surroundings. The microscope used for the analysis is an NX10 manufactured by Park Systems. Images were taken using non-contact mode. The images were processed using Gwyddion software. All tests were carried out under constant temperature conditions (25 °C). In the case of examining emulsion samples, the analysis procedure follows the description above.

Preparation of emulsions based on SAILs

Formulations containing the synthesized SAILs were prepared. The obtained emulsion samples were stored in tightly closed containers under constant temperature conditions (25 °C).

Method of preparation oil-in-water emulsion

The ingredients (sunflower oil, glycerol monostearate, cetyl alcohol) used to prepare the hydrophobic phase of the emulsion were heated to a temperature of 70 °C. During heating, the ingredients were stirred on a mechanical stirrer until completely melted. At the same time, citric acid and synthesized compound were dissolved in water. Once all the ingredients of the oil

phase were completely dissolved, the water phase was added to it while stirring on a mechanical stirrer.

Method of preparation water-in-oil emulsion

The first step in preparing a water-in-oil emulsion is analogous to that presented in *section 2.7.1*. However, in this case, ingredients (cetearyl alcohol, liquid paraffin, isopropyl palmitate, white petrolatum) were used to prepare the hydrophobic phase, which were also heated to a temperature of 70 °C. During heating, the ingredients were stirred on a mechanical stirrer until completely melted. Simultaneously, the hydrophilic phase was prepared, consisting of citric acid, a synthesized compound, and water. After dissolving the components, the water and oil phases were combined.

Characteristics of the emulsions

The emulsions prepared as described were analyzed by below mentioned ways.

organoleptic evaluation and determination of emulsion type

They were evaluated in terms of color, odor, and consistency (according to Polish Standard No. BN-77/6140-01). Furthermore, the type of emulsion formed was determined from the microscope Bresser LCD 50x-2000x. Whether it is an oil-in-water emulsion (o/w) or a water-in-oil (w/o) emulsion.

stability, size distribution and polydispersity index (PDI)

The stability assessment of the obtained formulations was conducted using a centrifugation test and zeta potential (ZP) analysis. In the centrifugation test, samples of the analyzed preparation are placed in a vial and subjected to centrifugation (Thermo Scientific *Heraeus Labofuge 300)*. As a result of this process, unstable preparations undergo stratification into their component phases. The samples are centrifuged for 60 minutes at speed 4000 revolutions per minute (rpm).

Moreover, ZP was determined from Smoluchowski's equation after measurements of electrophoretic mobility using a Litesizer DLS 500 (Anton Paar, Austria) at a stabilized temperature of 25 °C and neutral pH = 7.0. Testing of each emulsion took place in triplicate. Determination of droplet size distribution and PDI were carried out using Dynamic Light Scattering measurements in the same way as for ZP.

Rheological measurements

Rheological measurements were performed using a Physica MCR501 rotational rheometer (Anton Paar, Austria). The studies utilized a parallel plate system (diameter 60 mm, gap width 1 mm). Two types of experiments were conducted in oscillatory shear flow: strain sweep test and frequency sweep test. The measurements were carried out at a temperature of 25 °C.

Statistical analysis

The results in the study were calculated as the average from at least three independent experiments. Statistical significance of the differences between the average values was determined using variance analysis and the t-Student test, with differences considered significant at $p < 0.05$.

Computational methods

The Density Functional Theory (DFT) calculations were conducted using the Gaussian09 program [31]. The calculations were performed using the hybrid functionals B3LYP [32–34], cam-B3LYP [35], and ωB97x-D [36,37], with the basis set 6-311++G(d,p) and 6-31G(d,p) [38]. The Polarizable Continuum Model (PCM) with integral equation formalism variant (IEFPCM) method was employed to conduct calculations for solvents [39–41]. The initial geometry of the molecules was obtained for the cam-B3LYP/6-311++G(d,p) level of theory. Potential Energy Scanning (PES) was used to determine the position of aliphatic chains in the molecule. Potential energy calculations were performed for a series of rotamers to find the energetically favorable

conformations. The activation energy associated with the rotation of the aliphatic chain around the C-C and C-O bonds was determined. Dipole moment energies were calculated using several theoretical methods for various rotamers in vacuum and aqueous solution.
